# Inference of differential key regulatory networks and mechanistic drug repurposing candidates from scRNA-seq data with SCANet

**DOI:** 10.1093/bioinformatics/btad644

**Published:** 2023-10-20

**Authors:** Mhaned Oubounyt, Lorenz Adlung, Fabio Patroni, Nina Kerstin Wenke, Andreas Maier, Michael Hartung, Jan Baumbach, Maria L Elkjaer

**Affiliations:** Institute for Computational Systems Biology, University of Hamburg, Hamburg 22607, Germany; Department of Medicine, Hamburg Center for Translational Immunology (HCTI) and Center for Biomedical AI (bAIome), University Medical Center Hamburg-Eppendorf (UKE), Hamburg 20246, Germany; Institute for Computational Systems Biology, University of Hamburg, Hamburg 22607, Germany; Center for Molecular Biology and Genetic Engineering (CBMEG), State University of Campinas (Unicamp), Campinas, SP 13083-875, Brazil; Institute for Computational Systems Biology, University of Hamburg, Hamburg 22607, Germany; Institute for Computational Systems Biology, University of Hamburg, Hamburg 22607, Germany; Institute for Computational Systems Biology, University of Hamburg, Hamburg 22607, Germany; Institute for Computational Systems Biology, University of Hamburg, Hamburg 22607, Germany; Department of Mathematics and Computer Science, University of Southern Denmark, Odense 5000, Denmark; Institute for Computational Systems Biology, University of Hamburg, Hamburg 22607, Germany; Department of Neurology, Odense University Hospital, Odense 5000, Denmark; Institute of Clinical Research, University of Southern Denmark, Odense 5000, Denmark; Institute of Molecular Medicine, University of Southern Denmark, Odense 5000, Denmark

## Abstract

**Motivation:**

The reconstruction of small key regulatory networks that explain the differences in the development of cell (sub)types from single-cell RNA sequencing is a yet unresolved computational problem.

**Results:**

To this end, we have developed SCANet, an all-in-one package for single-cell profiling that covers the whole differential mechanotyping workflow, from inference of trait/cell-type-specific gene co-expression modules, driver gene detection, and transcriptional gene regulatory network reconstruction to mechanistic drug repurposing candidate prediction. To illustrate the power of SCANet, we examined data from two studies. First, we identify the drivers of the mechanotype of a cytokine storm associated with increased mortality in patients with acute respiratory illness. Secondly, we find 20 drugs for eight potential pharmacological targets in cellular driver mechanisms in the intestinal stem cells of obese mice.

**Availability and implementation:**

SCANet is a free, open-source, and user-friendly Python package that can be seamlessly integrated into single-cell-based systems medicine research and mechanistic drug discovery.

## Graphical Abstract

**Graphical Abstract:**
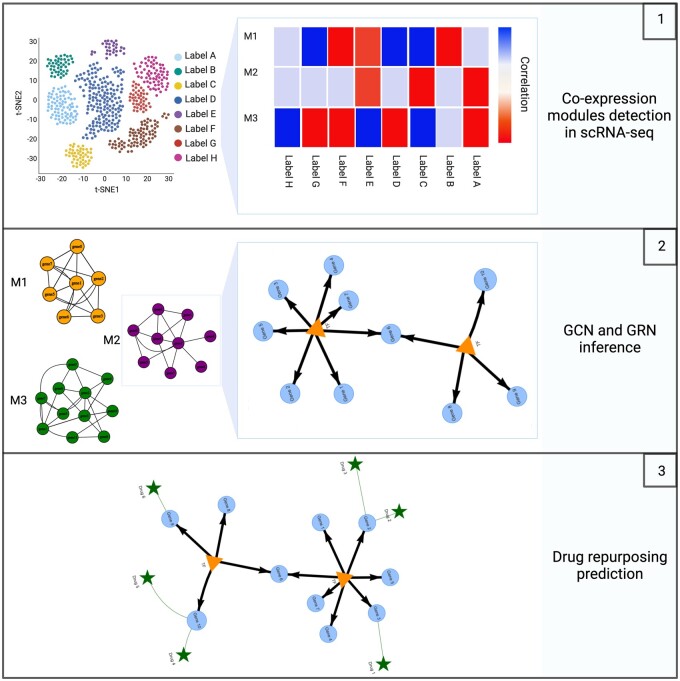

## 1 Introduction

The analysis of single-cell data is challenging, and currently, there is a lack of comprehensive computational tools that cover the entire process, from raw data analysis to the identification of disease driver mechanisms and the prediction of gene network-based drug (target or repurposing) candidates. Gene co-expression networks (GCNs) are increasingly utilized to investigate systems-level functionality and interplay of gene sets (Zhang and Horvath 2005). In these networks, genes are represented by nodes and associated with directed or undirected edges between the nodes based on similarity metrics of their expression patterns across cells or samples. Similar expression profiles (e.g. over time, across different experimental conditions, or along cellular differentiation trajectories) imply that the contributing genes are related functionally and may be regulated by a small set of (transcription) factors that drive their activity (Eisen *et al.* 1998). Just as mechanistic drivers and regulatory networks controlling genetic programs in different cell states, tissue types, developmental stages, and phenotypic states, the observable co-expression networks that represent these patterns are altered as well (Choobdar *et al.* 2019). Therefore, these co-expression networks approximate the driver mechanisms and their understanding is crucial for comprehending the complex molecular interplay between the regulatory genes underlying complex traits and diseases. Furthermore, co-expression analysis has a wide range of applications in genomics. For instance, it has been utilized in cell–cell interaction analysis based on ligand–receptor pair expression (Cabello-Aguilar *et al.* 2020), in the identification of disease mechanisms by analyzing co-regulated genes with known disease genes (Gov and Arga 2017), and in function prediction of uncharacterized genes based on their co-expression patterns with genes of known function (van Noort *et al.* 2003).

Existing tools for building GCNs typically begin by calculating correlation-based co-expression scores for each gene pair. They then identify gene modules, which are groups of significantly co-expressed genes. Integrating biological data and studying the network's topology is crucial for understanding the meaning of these co-expression interactions (Van Dam *et al.* 2018). Phenotypic associations, or the relationship between a phenotype and the expression profile of a module, are often used as a proxy for studying the molecular changes associated with a disorder (Gaiteri *et al.* 2014). Additionally, the scale-free topology property of GCNs allows for using graph theory metrics and methods to study these networks. Hub genes, or strongly linked genes within a gene module, play an important role in determining the functionality of a module (Pierson *et al.* 2015). Another common approach in co-expression analysis is to construct gene regulatory networks (GRNs), represented as directed graphs with two node types, transcription factors (TFs) and their target genes (TGs), and directed edges that model regulatory relationships (Emmert-Streib *et al.* 2014). When associated with disorders, those key regulatory networks can help identify potential drug targets that mitigate a particular dysregulated function.

In the case of single-cell omics data, each cell is treated as an individual sample, and the resulting modules can be associated with cell types or any available cell-level metadata. However, the increasing dimensionality of high-dimensional data derived from transcriptomic profiling at a single-cell resolution makes the task of GCN inference computationally intensive and challenging (Lähnemann *et al.* 2020). A way to address this issue is by pre-processing the data by creating “pseudo” bulk RNA-seq data. This is achieved by aggregating the read counts for each gene across a designated number of neighboring cells, reducing the data.

Numerous tools, such as COTAN (Galfrè *et al.* 2021), scLink (Vivian Li and Li 2021), and fcoex (Lubiana 2022), perform single-cell data co-expression analysis. However, they do not enable conversion of inferred GCNs to GRNs, where connections between genes have clear biological significance, for a comprehensive downstream analysis. Moreover, no tool covers the entire data analysis workflow, from scRNA-seq raw data to GCNs, GRNs, and drug repurposing candidates. To address these needs, we developed a Python package for single-cell co-expression network analysis (SCANet), which is available on PyPI and distributed under the MIT license. SCANet builds on a modified version of weighted gene co-expression network analysis (WGCNA) (Langfelder and Horvath 2008) for network inference and module detection. These modules are the subject of an extended analysis that includes trait and cell type associations, hub genes detection, regulatory relations by converting GCN to GRN, and drug repurposing candidate identification. SCANet includes a variety of descriptive visualizations to help users understand and analyze scRNA-seq data.

In summary, SCANet is a scRNA-seq profiling tool that first infers GCNs, extends them to GRNs, and identifies associated potential pharmacology targets and drug candidates to offer a deeper understanding and clinically actionable cellular mechanisms through a one-stop solution. While there are individual methods available for each step, SCANet offers an integrated approach that enhances our understanding of gene networks and potential drug targets. It brings together different pieces in a unique way, providing a tool to extract explainable GRNs and potential drug repurposing candidates from raw single-cell transcriptomics data.

In the remainder of this article, we will delve deeper into the implementation and functionality of the SCANet package. We will compare its capabilities with other related tools and demonstrate its power by analyzing scRNA-seq data from peripheral blood mononuclear cells (PBMCs) of patients with acute respiratory illness (Cillo *et al.* 2021) and from the enteroendocrine linkage in the small intestine of diet-induced obese mice (Aliluev *et al.* 2021).

## 2 Materials and methods

### 2.1 The concept of the SCANet method

**Figure 1.**
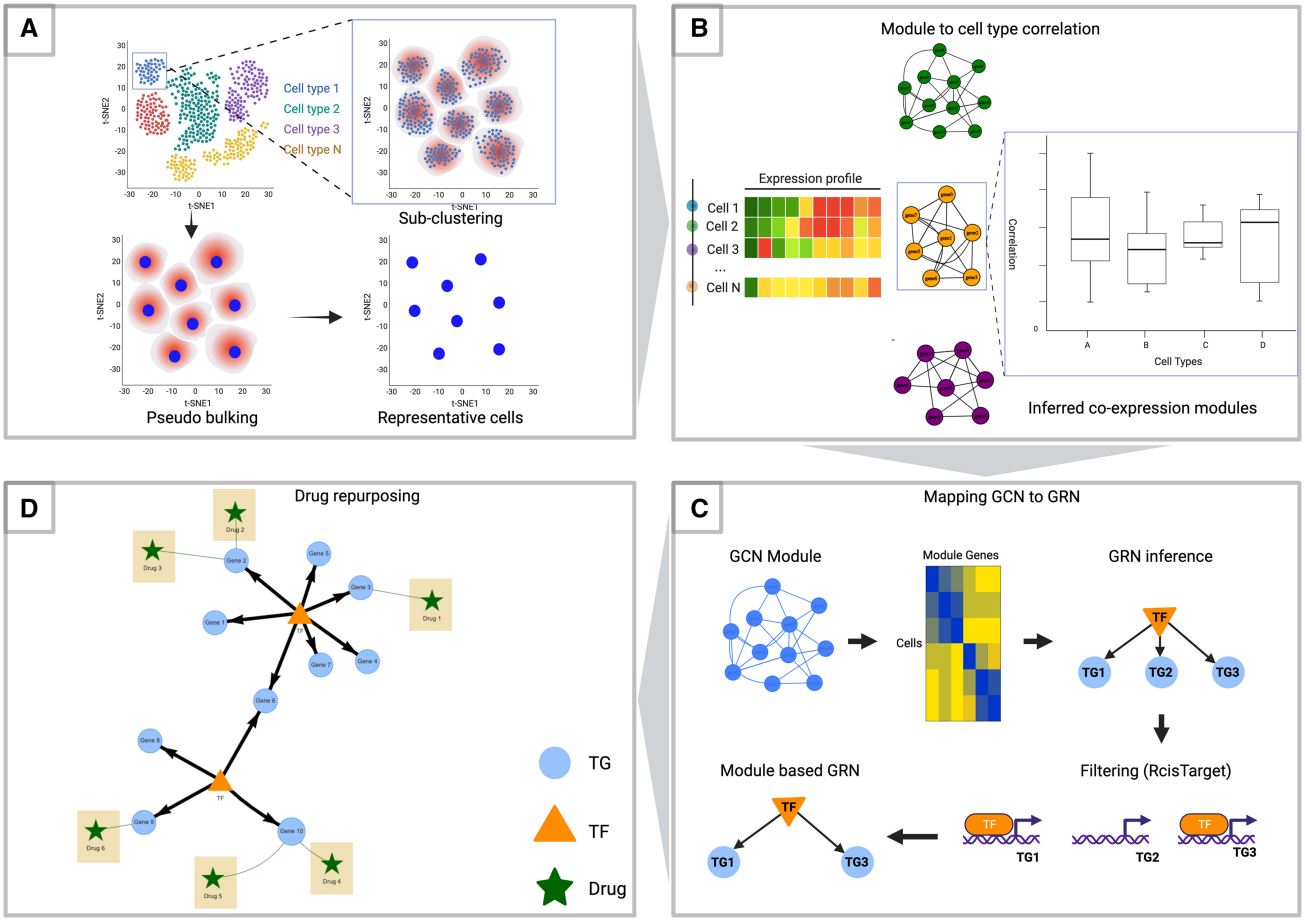
SCANet workflow. The pre-processed scRNA-seq count matrix serves as input for the workflow. (A) Representative cells are formed by clustering cells from each cell type into *N* sub-clusters. (B) Dropping cell-type annotation allows for a *de novo* inference of co-expression modules that are related to cell type or any cell-level metadata. (C) GCN to GRN conversion is accomplished by inferring GRN from the module’s genes, RcisTarget database is used to filter false positives by examining the TF-binding affinity. (D) The predicted networks of TFs and their TGs, along with potential drug repurposing candidates, can be visualized all together in one combined network.

The proposed value chain for scRNA-seq data gene co-expression-based analysis starts by giving preprocessed data in the form of an expression matrix as input. Each row represents a cell and each column represents a gene. The data are accompanied by additional cell-level metadata such as cell type, source, and batch. The preprocessing step and parameter selection are left to the user as they are highly dependent on sequencing technology, data quality, and experimental design. After importing, the process begins by grouping neighboring individual cells to create representative cells. This step is implemented to address the high-resolution and sparse nature of the data, where each cell type is represented by thousands of cells and their respective expression profiles across thousands of genes. All cells from each cell type are then further divided into *N* sub-clusters. To downsample the data, a single representative cell is created for each sub-cluster by averaging the gene expression profiles of all the cells within the sub-cluster (Fig. 1A). Next, the WGCNA algorithm is utilized to infer GCNs, which are organized as modules of genes that exhibit strong co-expression patterns. SCANet allows users to explore and visualize GCNs in various ways, such as by correlating them with cellular metadata, identifying key network hubs, visualizing drugs targeting genes within the module, and checking for associations with specific diseases or biological pathways (Fig. 1B). This analysis aims to identify specific modules of interest for further downstream analysis. We employ a multi-step approach to effectively assign biologically relevant relationships to the inferred GCNs and eliminate inaccuracies caused by false gene–gene correlations (e.g. noise induced and randomly observed). We map the GCN modules of interest by scanning for TF genes and feeding them into the GRN inference function using GRNBoost2, an algorithm for regulatory network inference using gradient boosting, based on the GENIE3 architecture (Moerman *et al.* 2019). Next, we use RcisTarget (Aibar *et al.* 2017) to ensure accuracy by performing cis-regulatory TF-binding motif enrichment analysis. The remaining edges that are connected to TGs within the scRNA-seq data construct the GRN, a directed regulatory graph (Fig. 1C). The SCANet framework streamlines the drug repurposing process by providing a function for exploring potential drug candidates targeting identified small GCNs and GRNs. This is achieved by integrating the human interactome with various drug–target interaction databases using the Drugst.One package (Maier *et al.* 2023). The resulting predicted networks, candidate drug targets, and putative drug repurposing candidates can be visualized in a single comprehensive network (Fig. 1D).

### 2.2 Input data pre-processing

Input data can be any scRNA-seq data format (e.g. HDF5, H5ad, or loom) that can be read by Scanpy (Wolf *et al.* 2018). Once the data are loaded and stored as an expression matrix, each row represents a cell and every column represents a gene. Each entry in the matrix represents the expression level of a particular gene in a given cell. The inputted data are usually accompanied by extra metadata describing cells such as cell type, source, and batch. We have integrated the essential pre-processing functions from Scanpy to make it possible to perform quality control, normalization, data correction, feature selection, dimensionality reduction, and visualization. The pre-processing step and parameter selection are up to the user’s discretion, as they are highly dependent on the technology used during the sequencing, data quality, and the desired experimental design.

### 2.3 Representative cells

ScRNA-seq data are high-resolution—meaning every cell type is represented by hundreds or even thousands of cells and their respective expression profile across thousands of genes. Moreover, many of the analyses require biological replicates of experimental conditions to assess statistical significance. In SCANet, we circumvent these problems by creating representative cells through the grouping of neighboring single cells. In this process, the data from every single cell in a group of neighboring cells are combined into a single sample that represents the overall signal across these cells. The cells for each cell type are sub-clustered into *N* sub-cluster (Supplementary File 1 provides a user guideline outlining best practices for selecting appropriate *N* values) and each sub-cluster is turned into a single representative cell by averaging the gene expression profiles across its cells (Fig. 1B). The core assumption behind this procedure is that the individual cells grouped together are sufficiently similar that we are uninterested in understanding their differences.

### 2.4 Inference and functional analysis of co-expression modules

Using the down-sampling method procedure described above, we significantly reduce the number of cells. The cell types now are represented with the same number (*N* same number of selected sub-cluster) of the representative cells (Fig. 1B). We exploit the R framwork BioNERO (Almeida-Silva and Venancio 2022), where the GCN inference employs the WGCNA algorithm, which is implemented in the WGCNA R package (Langfelder and Horvath 2008). Through utilizing Pearson’s *r*, Spearman’s, or biweight midcorrelation (median-based, which is less sensitive to outliers), a matrix of pairwise gene–gene correlations can be constructed. To accentuate differences, the matrix is transformed into an adjacency matrix. By applying a threshold to continuous co-expression correlation values and converting them into binary connections, this procedure highlights strong associations while disregarding weaker ones. Signed, signed hybrid, or unsigned GCNs can all be inferred by users, and the type of the network has an impact on how adjacency matrices are computed. Positive and negative correlation coefficients are interpreted differently in signed networks (the default), because they preserve the correlation signs. Only positive correlations are taken into account in signed hybrid networks since all negative correlation coefficients are treated as zero. Unsigned networks consider the absolute value of the correlation coefficients.

The GCN inferred is structured as gene modules displaying strong patterns of co-expression. The next step in the proposed workflow is to analyze these modules to further designate candidates for the downstream analysis. Users can first correlate these modules to the cell’s metadata such as cell type or phenotype thus identifying modules whose expression levels increase or decrease in a particular condition (Fig. 1C). The “Modules selection guidelines” section in Supplementary File 1 offers a comprehensive description of recommended guidelines for the selection of modules. Additionally, users can identify network hubs by looking for the intersection of the top 10% of the most connected genes in the module. Furthermore, the modules of interest as a whole or reduced sub-module based on gene correlation can be visualized as a network. Users have the choice to also visualize drugs targeting the genes within this module. They can also check whether the module graph fits the scale-free topology, a characteristic of real-world biological networks (Barabási *et al.* 2003).

### 2.5 Converting GCN to GRN

In contrast to GRN, in GCN the direction and type of inferred co-expression relationships are not determined. In GRN, a directed edge connecting two genes represents a real biochemical process such as activation or inhibition (Roy *et al.* 2014). In this workflow, we mapped the GCN modules of interest (i.e. retained modules following the functional analysis of the GCN modules described above) by scanning for TFs genes among the module’s genes. These TFs and the processed scRNA-seq data are fed to the GRN inference function. GRNBoost2 (Moerman *et al.* 2019) was adopted to construct GRN. GRNBoost2 is a regression-based GRN inference method and based on the same architecture as GENIE3 (Huynh-Thu *et al.* 2010). GRNBoost2 is a faster alternative to GENIE3 due to its scalability, thus paving the way for network inference from large datasets (Pratapa *et al.* 2020). GRNBoost2 builds a tree-based regression model for each gene in the database with the aim of predicting the expression profile of the TG in the function of the expression profile of candidate TFs. The regulators whose expression profiles largely contribute to predicting the TG, are retained as candidate edges in the resulting GRN. Additionally, to exclude potential false prediction, we used the RcisTarget (Imrichová *et al.* 2015, Van de Sande *et al.* 2020) database to perform cis-regulatory TF-binding motifs enrichment analysis of each TFs and their TGs. RcisTarget validates for every edge (TF, TG) in the GRN, if the regulatory binding motif for the TF is significantly enriched at the cis-regulatory region of the TG, otherwise it is discarded as a potential regulator of this TG. The remaining edges construct the GRN which is a direct graph (i.e. TF pointing to the gene orientation indicates regulation direction). This GRN construction procedure (i.e. GRNBoost2 followed by RcisTarget) was adopted from pySCENIC workflow (Van de Sande *et al.* 2020).

### 2.6 Association of drug target

Drug repurposing is a useful strategy that gives new treatment alternatives by identifying possible uses for already-approved drugs, a process that can take years in the creation of vaccines and pharmaceuticals (Sun *et al.* 2017). SCANet framework offers a function that enables users to easily explore potential drug repurposing candidates targeting an identified mechanistic key driver network. To this end, we integrated the Drugst.One package, an open, community-driven package to close the gap between mechanism mining and mechanistic drug repurposing prediction.

Drugst.One accesses multiple databases to construct large, heterogeneous networks, which it utilizes to retrieve information based on the context of various domains. To connect the human interactome with drug–target interaction databases, Drugst.One begins with a protein–protein interaction network and maps the proteins to a primary common identifier (UniProt ID), as well as other ID spaces such as Entrez and Ensembl. This mapping allows Drugst.One to link the respective drug targets in databases such as NeDRex (Sadegh *et al.* 2021), DrugBank (Wishart *et al.* 2018), DrugCentral (Avram *et al.* 2021), ChEMBL (Davies *et al.* 2015), and DGIdb (Freshour *et al.* 2021). Consequently, drugs can be linked to proteins, forming a comprehensive heterogeneous network.

SCANet uses by default the database “NeDRex,” which is an extensive dataset encompassing small molecules, biologics, approved drugs, and experimental ones by merging data from DrugBank and DrugCentral. However, to provide more flexibility, SCANet offers the option to select any available data source, enabling users to choose one that aligns with their specific analysis requirements and preferences. This flexibility also extends to the information about the effects of drugs on proteins, which may not be available in all resources. A list of available datasets can be found here: https://drugst.one/doc#implementation_datasources.

## 3 Results

### 3.1 SCANet provides more functionalities than existing single-cell analysis tools

The quick brown fox jumps over the lazy dog. We compared SCANet to existing software that offer scRNA-seq analysis based on co-expression, namely COTAN (Galfrè *et al.* 2021), scLink (Vivian Li and Li 2021), and fcoex (Lubiana 2022). These packages were compared based on the similarity of the provided functionalities (Table 1). SCANet—in contrast to existing scRNA-seq co-expression analysis packages—provides an end-to-end analysis platform that allows users to not only perform co-expression analysis but also additional downstream analyses. This helps to obtain a better mechanistic understanding of the inferred co-expression networks by uncovering their regulatory programs and by identifying potential drug candidates targeting their mode of action.

**Table 1.** Functionality level comparison of the current existing tools for single cell co-expression analysis.

	Data pre-processing	Modules analysis	GCN to GRN conversion	Network visualization	Drug target prediction
SCANet	✔	✔	✔	✔	✔
COTAN	✔	✗	✗	(✔)	✗
scLink	✔	✗	✗	✗	✗
fcoex	✔	✔	✗	✔	✗
ASGARD	✗	✗	✗	✗	✔
scDrug	✔	✗	✗	✗	✔

✔ has the function.

✗ lacks the function.

### 3.2 Monocytic gene regulatory networks related to mortality of acute respiratory illness

**Figure 2.**
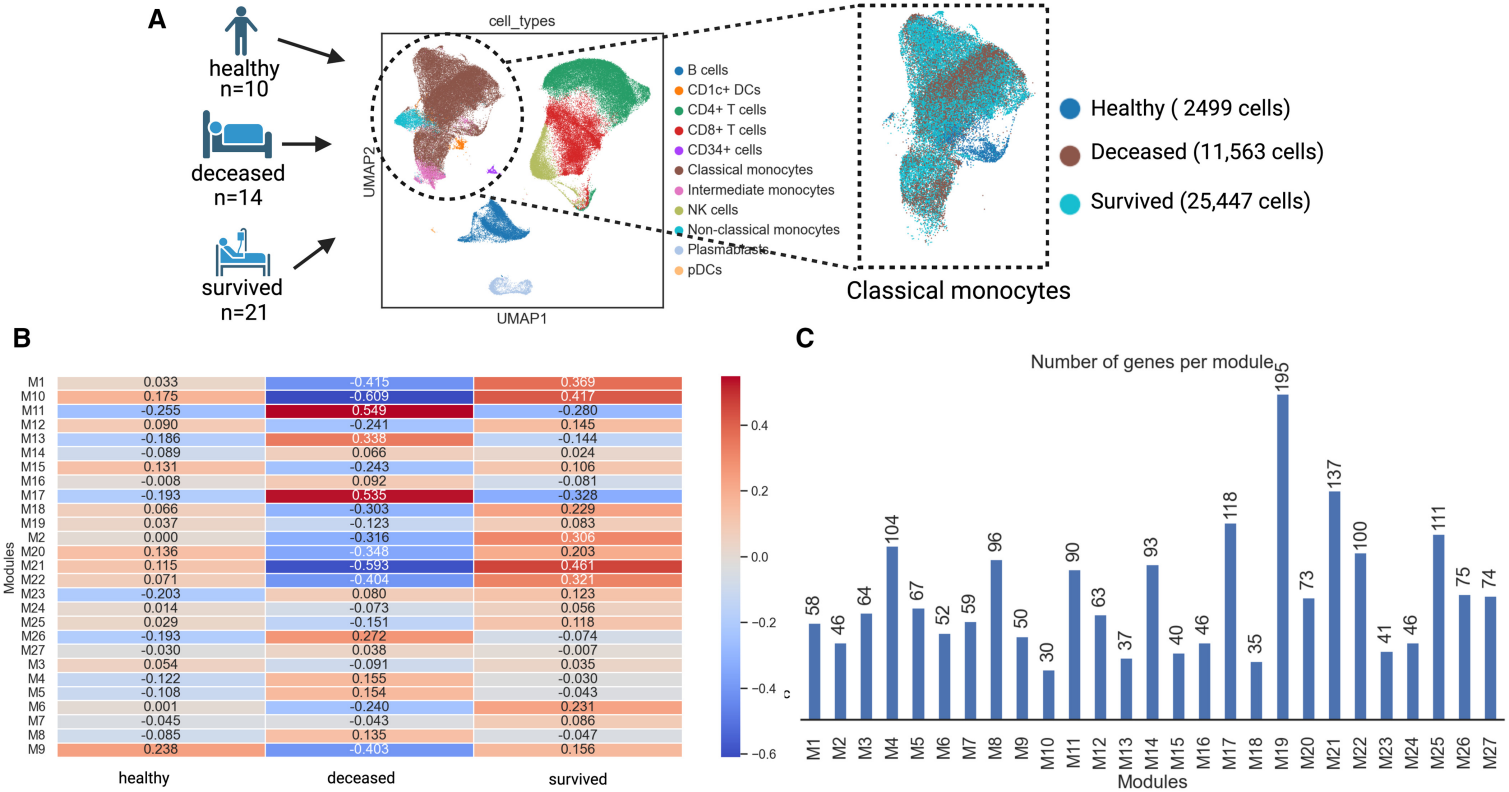
Co-expression modules in classical monocytes of patients with acute respiratory illness. (A) Selection of classical monocyte sub-population from PBMCs of patients who died or survived acute respiratory illness as well as healthy controls. (B) The 27 co-expressed gene modules in classical monocytes across deceased, survived, and healthy individuals color coded based on Spearman’s correlation. (C) The number of co-expressed genes in each module.

In transcriptional PBMC profiles of patients who died or survived acute respiratory illness, the classical monocyte subpopulation had (i) the highest viral load, (ii) distinctive profiles predictive of disease mortality, and (iii) a unique transcriptional signature while viral infection (Cillo *et al.* 2021). We used SCANet to explore and potentially explain how differential GRNs of classical monocytes are related to mortality in acute respiratory illness using the processed scRNA-seq data from Cillo *et al.* (2021) (Fig. 2A). We detected 27 co-expression gene modules of classical monocytes among healthy, deceased, and surviving patients (Fig. 2B) with 30–195 genes per module (M) (Fig. 2C). We have provided all the inferred co-expression modules and their corresponding genes in Supplementary File 3 for better clarity and to assist in further investigations. In particular, M11 had the highest positive correlation with deceased patients, while M21 had a high correlation with surviving patients (Fig. 2B).

**Figure 3.**
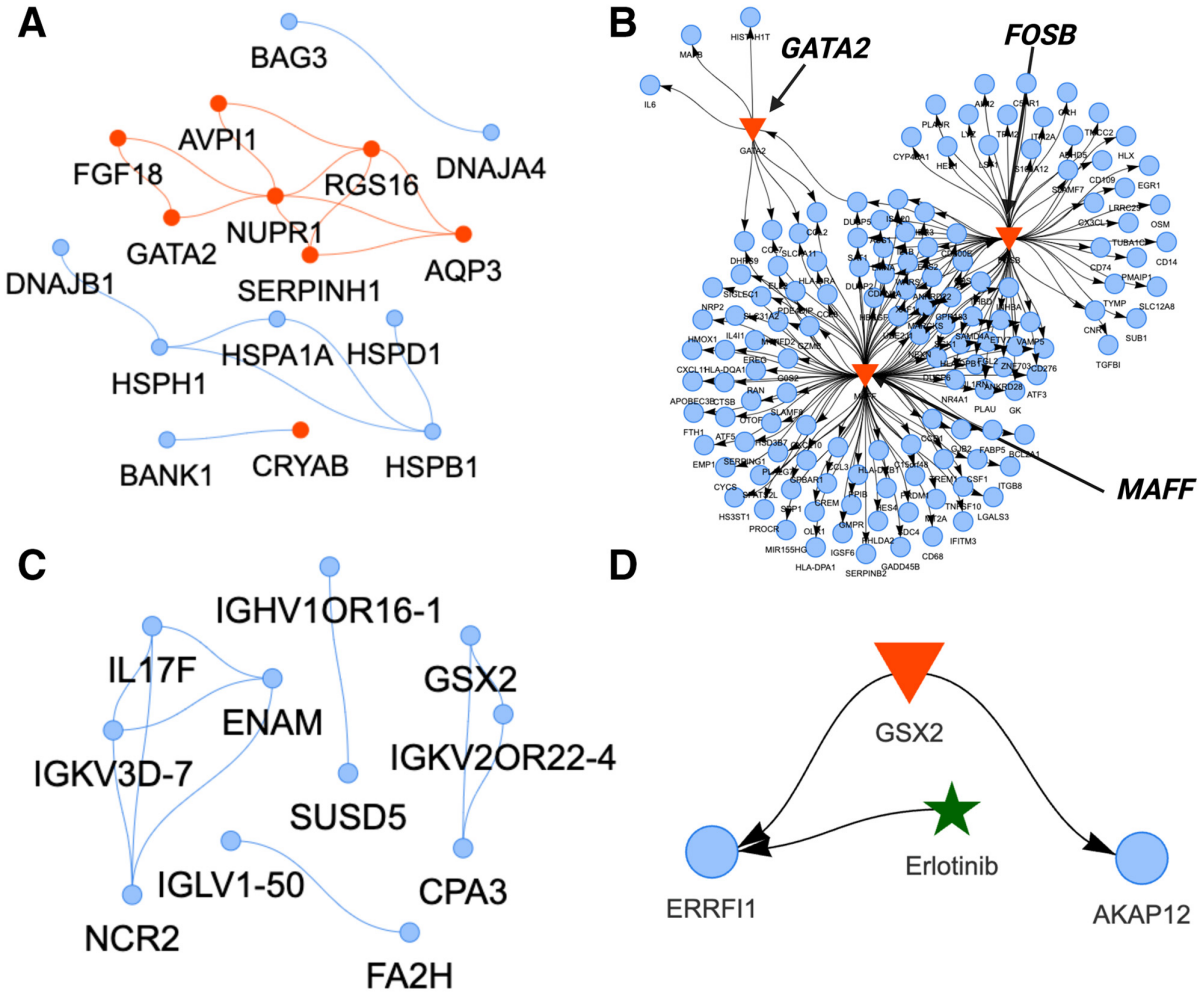
Gene co-expression and regulatory networks (GCN, GRN) of selected gene modules in classical monocytes associated with survival or mortality of patients with acute respiratory illness. (A) The GCN (blue nodes) of the gene module associated with deceased patients (correlation ≥ 0.9). Red nodes indicate hub genes. (B) The GRN of the same module with three major TFs (*GATA2*, *MAFF*, *FOSB*) (red triangles) regulating their TGs in the module (blue nodes). (C) The GCN and (D) the GRN of the gene module associated with surviving patients.

In the GCN of M11 (Fig. 2B, 16 out of 90 genes exhibit correlation ≥ 0.9), over half of the genes and hubs code for heat shock proteins (e.g. *HESPB1*, *DNAJA4*, *CRYAB*) (Fig. 3A), mirroring the systemic innate stress response against severe COVID19 infection (Danladi and Sabir 2021). In the context of COVID-19, activated heat shock responses are insufficient in resolving virus-induced inflammatory bursts, potentially contributing to a fatal cytokine storm (Heck *et al.* 2020). The GRN of M11 also indicates the production of cytokine storms (Fig. 3B).

Three TFs (*GATA2*, *MAFF*, *FOSB*) in the GRN regulate expressed TGs of highly pro-inflammatory reactions (Table 2 and Fig. 3B). These cytokine patterns are predictive of COVID-19 mortality independently of demographics and comorbidities (Del Valle *et al.* 2020). The GRN TGs of chemokine ligands (CCLs, CXCLs) are mostly secreted from monocytes in COVID-19 (Singh *et al.* 2022) (Table 2). Elevated levels of tumor necrosis factor (TNF) and interleukins (ILs) in the GRN are also risk factors for disease severity and mortality in COVID-19 (Del Valle *et al.* 2020, Siddhuraj *et al.* 2021). The activation of complement factors (Table 2) in COVID-19 is caused by recognizing the spike and nucleocapsid of SARS-CoV-2 directly via the lectin pathway or indirectly via antibody-mediated recognition through the classical pathway (Niederreiter *et al.* 2022). Additionally, the matrix metalloproteinases (MMPs) cause extracellular tissue damage, the TFs of interferon regulatory factors (IRFs) trigger additional pro-inflammatory genes, and high expression of antigen-presenting genes induce adaptive immunity (Table 2). These diverse accelerated immune mechanisms can be responsible for the fatal outcomes. Targeting one pathway may stimulate downstream compensatory immune responses due to the complexity of the inflammatory network of classical monocytes. Still, here we discovered a combination of three potential regulators that may ensemble an enhanced inflammatory response.

**Table 2.** TGs of *GATA2*, *MAFF*, and *FOSB* in GRN of deceased patients responsible for excessive release of pro-inflammatory signals.

Molecular function	Genes
Complement	*C1QA*, *C1QBP*, *C1QC*, *C5AR1*, *FCN1*
Chemokine ligands	*CCL2*, *CCL3*, *CCL4*, *CCL7*, *CCL8*, *CX3CL1*, *CXCL10*, *CXCL11*, *CXCL16*, *CXCL2*, *CXCL3*, *CXCL5*, *CXCL6*
Chemokine receptors	*CCR1*, *CCR7*, *CCRL2*
Antigen presenting	*HLA-DPA1*, *HLA-DPB1*, *HLA-DQA1 HLA-DRA*, *HLA-DRB1*, *HLA-DRB5*, *HLA-DPA1*, *CD83*, *CD74*, *CD40*
Immune activation	*CD7*, *LYZ CD72*, *CD68*, *TREM1*, *TREM2*, *ICAM*, *CD14*
Cytokine production	*CD38*, *CSF1*, *CSF2*, *CST3*
IL ligands and receptors	*IL6*, *IL7R*, *IL1B*, *IL1RN*, *IL32*, *IL10*, *IL4I1*
IRFs	*IRF4*, *IRF7*, *IRF8*
TNF signaling	*TNF*, *TNFAIP6*, *TNFRSF21*, *TNFRSF4*, *TNFSF10*
Breakdown of extracellular structures	*MMP1*, *MMP19*

Given the network’s association with fatal COVID-19 outcomes, we explored potential drugs for therapeutic repurposing (Supplementary File 4). Specifically, we identified anti-inflammatory agents like amlexanox, dexamethasone, and olopatadine, which could modulate these aggressive immune responses. Additionally, the antiviral drug, abacavir, can potentially counteract the virus directly (Supplementary File 4). These combi-drugs may target and temper the complex inflammatory network interactions observed in these critically ill patients.

The M21 co-expressed gene module (Fig. 2B, 11 genes out of 137 genes exhibit correlation = 1.0) of classical monocytes in the surviving patient group had a more lung-directed phenotype. This is indicated in the GCN by markers such as carboxypeptidase A3 (*CPA3*) which participates in lung tissue homeostasis and inflammatory remodeling (Siddhuraj *et al.* 2022), and the interleukin 17F (*IL-17F*) that orchestrates the immune system of the resident lung monocytes/macrophages (Ferreira *et al.* 2020) (Fig. 3C). The transcriptional regulator in the GRN was the *GSX2*, a known regulator of cell fate (Waclaw *et al.* 2009) (Fig. 3D). It controls two TGs (Fig. 3D): (i) *ERRFI1* (*MIG6*), a lung marker (Jin *et al.* 2009) that also works as an immediate early response gene to inhibit proliferation and invasion (Li *et al.* 2014); and (ii) *AKAP12* which restricts and regulates inflammation at tissue sites (Cha *et al.* 2014). To identify repurposable drugs, we examined GRN–drug interactions (Fig. 3D). Erlotinib, primarily known for EGFR inhibition in cancer therapy, also interacts with ERRFI1. While its primary function involves EGFR and growth inhibition, its effect on ERRFI1 remains uncertain. Since ERRFI1 inhibits EGFR, an ERRFI1 agonist could enhance anti-proliferative and anti-invasion effects. Additionally, lipid agonists like LPA and S1P, and glucocorticoids such as hydrocortisone, can modulate ERRFI1’s activity (Descot *et al.* 2009, Mojica *et al.* 2020). The precise effect should be examined further experimentally.

In summary, by using SCANet, we discovered small distinctive GRNs of a pro-inflammatory response in classical monocytes that are associated with mortality. Whereas in surviving patients, the classical monocytic networks may have acquired a more lung-specific phenotype to inhibit invasion, limit excessive inflammation, and initiate tissue remodeling.

### 3.3 Obesogenic enriched stem cell GCN, GRN, and drug candidates to reverse intestinal maladaptation

**Figure 4.**
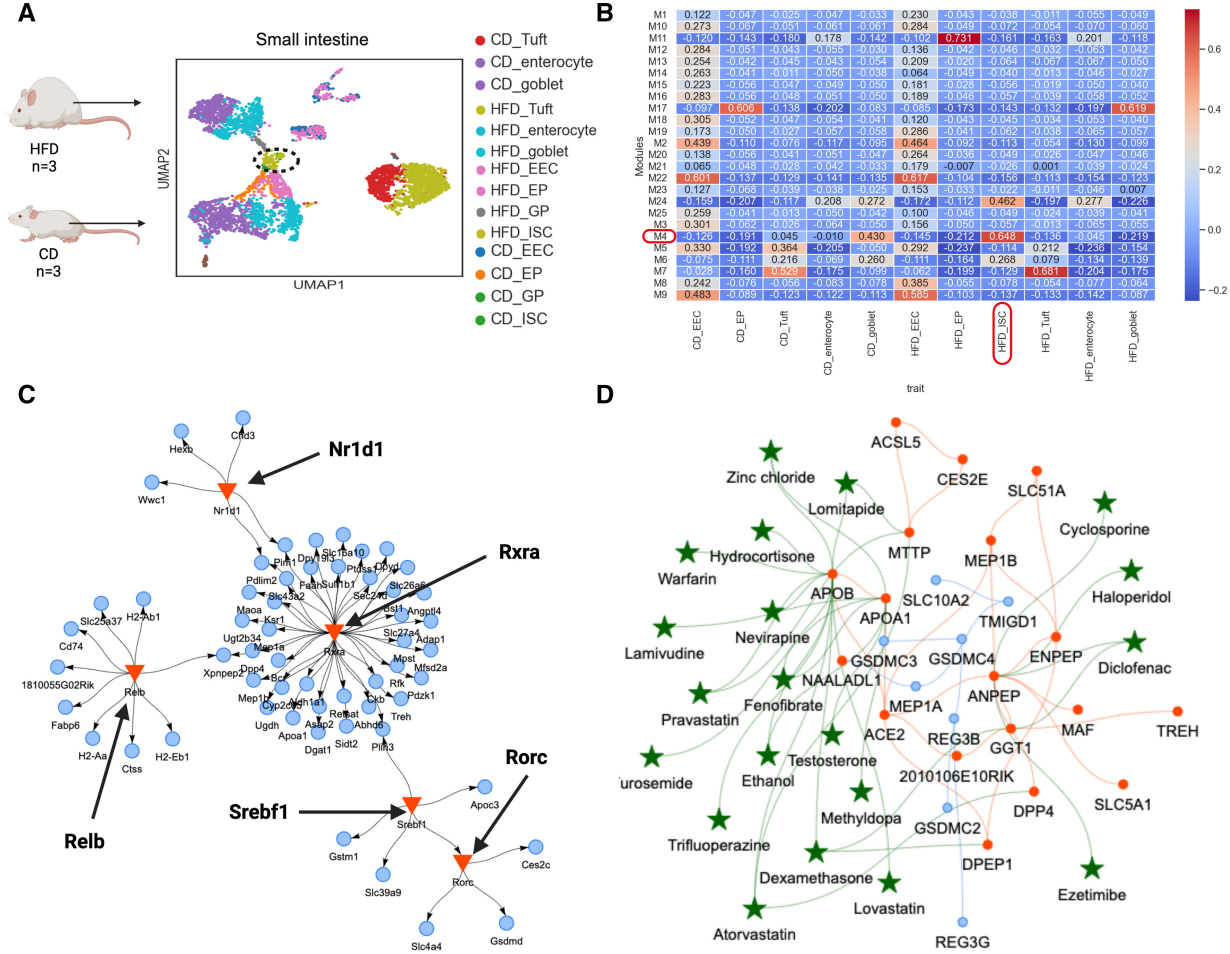
GCN, GRN, and repurposing drug candidates to target ISCs in isolated villi of diet-induced obesity. (A) scRNA-seq data from villi of the small intestine from mice on high-fat/high-sugar diet (HFD) or CD. ECC, enteroendocrine cells; EP, enterocyte progenitors; GP, goblet progenitors; ISC, intestinal stem cells; Tuft, Tuft cells; enterocyte, enterocytes; goblet, goblet cells. (B) The 25 co-expression gene modules retrieved between cell types of the enteroendocrine lineage. The gene module with the strongest unique correlation to HFHSD intestinal stem cells (ISCs) is highlighted. (C) The GCN of M4 (correlation ≥ 0.8, blue nodes) including hub genes (red nodes) in HFHSD ISCs with interacting repurposable drugs (green stars). (D) The GRN of the same module is regulated by four major metabolic transcriptional factors (RXRA, NR1D1, SREBF1/SREBP-1, RORC) (red triangles) connected to their TGs (blue nodes).

High-fat/high-sugar diet (HFHSD) causes proliferation of intestinal stem cells (ISCs) leading to cell fate changes, increased villus length in the small intestine and altered cell composition (Aliluev *et al.* 2021) (Fig. 4A). This results in functional maladaptation of the gut and increased risk of metabolic syndrome and gastrointestinal cancer (Aliluev *et al.* 2021). With SCANet, we identified 25 co-expressed gene modules between cell types of the enteroendocrine lineage from isolated villi in HFHSD and control diet (CD) mice (Fig. 4B). We have provided all the inferred co-expression modules and their corresponding genes in the Supplementary File 5 for better clarity and to assist in further investigations. The GRN of the gene module with the strongest correlation to ISC in HFHSD (M4) revealed a genetic program controlled by four TFs (RXRA, NR1D1, SREBF1/SREBP-1, and RORC), all known regulators of lipid metabolism (Fig. 4C) (Crewe *et al.* 2019, Hunter *et al.* 2021, Pan *et al.* 2022, Zou *et al.* 2022). To explore potential drug targets specific for altered ISC mechanisms that underlie intestinal maladaptation and contribute to the development of obesity and prediabetes (Aliluev *et al.* 2021), the same module was selected for GCN–drug interactions (correlation ≥ 0.8) (Fig. 4D). Drugs reducing cholesterol and abnormal lipid levels (lovastatin, fenofibrate, lomitapide, pravastatin, atorvastatin), as well as anti-hypertensive drugs (methyldopa, furosemide, warfarin), interact with hub genes of fatty acid transporters (*APOB*, *APOA1*, *MTTP*) and the *DPP4* gene, associated with type 2 *diabetes mellitus* (Röhrborn 2015). Additionally, Ezetimibe, which is used to treat lipid abnormalities, was connected to the gastric membrane protease *ANPEP* in the ISC of HFHSD mice (Fig. 4D).

With SCANet, we identified a unique co-expression-enriched obesogenic network in ISCs. Multiple hub genes were connected to potentially repurposable drugs known to treat lipid abnormalities implying multiple candidate treatment options to reduce the high proliferative ISCs for preventing diet-induced intestinal maladaptation and prediabetes (Aliluev *et al.* 2021) as well as potential obesity-induced carcinogenesis (Pourvali and Monji 2021).

## 4. Discussion

In recent years, the rapid advancement of scRNA-seq technologies has led to the development of various methods for the analysis of scRNA-seq data, including the widely adopted approach of inferring GCNs. However, it is important to note that GCNs are limited in their representation of gene–gene correlations and do not necessarily imply a biological relationship such as regulation or potential modulation—limiting their utility for the identification of clinically actionable mechanisms. SCANet overcomes these limitations. It is the first all-in-one tool for scRNA-seq data analysis (a tutorial and an example are also provided in Supplementary File 2), which integrates co-expression analysis with other analytical approaches such as GRN reconstruction, hub gene identification, module annotation, and mechanistic drug repurposing. The conversion of GCNs to GRNs to infer biologically relevant gene regulation reduces noise and the risk of false-positive findings. However, it may also lead to an increased risk of false negatives, causing some subtle true relationships between genes to be undetected. This limitation should be noted when interpreting the outcomes of these analyses. SCANet employs cutting-edge algorithms to streamline the workflow from data acquisition to analysis and interpretation. SCANet extracts co-expression modules prior to cell annotation, which allows for the identification of true biological variations shared or unique between two or more cell types or phenotypes. SCANet offers unique functionalities compared to related tools. It allows for a deeper understanding of cellular mechanisms by uncovering *de novo* regulatory relationships, key drivers of specific phenotypes/disease types, and therapeutic targets for potential repurposable drug candidates. SCANet can effectively extract biologically relevant signals from scRNA-seq data, as demonstrated by the identification of pro-inflammatory regulatory networks in classical monocytes associated with mortality in patients with acute respiratory illness, and the repurposing of drug candidates targeting a metabolic network in highly proliferative ISCs of diet-induced obese mice.

### 4.1 Limitations

Firstly, GCNs rely on correlation, not causation, potentially leading to false positives in co-expression analysis. In SCANet, the goal is to reduce false positives by converting GCNs into GRNs, ensuring that edges represent biologically meaningful relationships between genes. Secondly, gene–drug databases in Drugst.One update weekly, impacting reproducibility. Although old results are accessible, running identical analyses and parameters on newer datasets may yield different results. To tackle this, a version control system is being developed for Drugst.One’s upcoming release, ensuring consistent results within SCANet across versions. Thirdly, gene–drug or gene–gene interactions are context-dependent, influenced by tissue specificity, and may not apply universally. Many drugs have multiple targets, and their effects result from a combination of interactions. Validating drug candidates can address these issues. Nevertheless, SCANet remains a robust tool for exploring scRNA-seq data, enabling the discovery of biologically relevant signals, regulatory relationships, and potential drug repurposing targets.

## Supplementary Material

btad644_Supplementary_Data

## Data Availability

This article analyzes existing, publicly available data: Cillo covid19 processed data (Cillo *et al.* 2021) GSE180578 and Villus processed data (Aliluev *et al.* 2021) GSE147319. SCANet is publicly available as a Python package on Github: https://github.com/oubounyt/SCANet and pypi: https://pypi.org/project/scanet/. A tutorial and an example are also provided in this article’s Supplementary File 2.
