# Supplementary material for: Inference of differential key regulatory networks and mechanistic drug repurposing candidates from scRNA-seq data with SCANet
Source: Bioinformatics. 2023 Oct 20;39(11):btad644. doi: 10.1093/bioinformatics/btad644 (PMC10628438; doi:10.1093/bioinformatics/btad644)
Supplement: btad644_Supplementary_Data [file btad644_supplementary_data.zip › Supplementary file 1.pdf]

**Parameter selection guidelines:**

The determination of the optimal number of subclusters 'N' during the data downsampling step is influenced by various key factors associated with the scRNA-seq dataset being analyzed. To provide guidance in this decision-making process, we offer the following recommendations, which are contingent on different data-driven scenarios:

**Small 'N' (e.g., 3 or 5):**
If you choose a very small 'N', say 2 or 3, for each condition, your representative samples will be extremely small and might not accurately represent the underlying biological variability. You might end up with high variability and noisy results. You might not capture the full biological complexity, and the results could be less reliable.

**Moderate 'N' (e.g., 10-25):**
Choosing a moderate 'N' could provide a better balance between capturing biological variability and computational efficiency. With more cells in each representative sample, you're more likely to capture representative expression profiles and achieve more robust statistical results.

**Large 'N' (e.g., > 50):**
Opting for a large 'N' would increase the statistical power of your analysis. The larger sample size would allow you to better estimate population-level expression profiles, reducing the impact of cell-to-cell variability. However, extremely large representative samples could lead to oversmoothing and might obscure rare or cell-type-specific signals.

**Impact of 'N' on Analysis Outcome:**

**Statistical Power:**
Larger 'N' generally increases statistical power. With more cells in each representative samples, you can detect subtle changes in gene expression more reliably, especially if the changes are consistent across cells.
**Biological Variability:**
Smaller 'N' might not capture the full biological variability present in the dataset, potentially leading to false negatives or missing important biological insights. Larger 'N' might homogenize the expression profiles and mask cell-to-cell variability.
**Computational Resources:**
Larger 'N' can increase the computational resources (memory and processing time) required for analysis. Smaller 'N' would be computationally faster but might not yield robust results.
**Recommendation:**
Based on the considerations above, we recommend carefully evaluating the characteristics of your specific dataset before determining the most suitable 'N'. It's important to strike a balance between

capturing biological insights and maintaining computational efficiency. Conducting sensitivity analyses with different 'N' values can provide valuable insights into the stability and consistency of your results.

**Note:** If the value of 'N' exceeds the number of cells in a cluster(s) this cluster(s) will be excluded from the analysis. The user will receive a warning generated by the function in such cases.

**Modules selection guidelines:**

In SCANet analysis, the number of co-expression modules returned by the analysis is usually too large for comprehensive downstream analysis. Therefore, users need to select specific modules of interest. We suggest different approaches to achieve this:

Gene Module Inspection: The first approach involves inspecting gene modules to identify genes or markers of interest. Subsequently, users can perform downstream analysis on the modules that contain these genes.

Correlation and Annotation: The second recommended approach is to consider the correlation of modules with annotations. If a module exhibits a significant high correlation (>= 0.5) or low correlation (<= -0.5) under the condition of interest (e.g., diseased), but it shows an opposing correlation or no correlation (around 0) with another condition (such as healthy), it is worth considering for downstream analysis.

Utilizing these strategies, users can effectively narrow down the number of modules for in-depth downstream analysis using SCANet.
