# Supplementary material for: Inference of differential key regulatory networks and mechanistic drug repurposing candidates from scRNA-seq data with SCANet
Source: Bioinformatics. 2023 Oct 20;39(11):btad644. doi: 10.1093/bioinformatics/btad644 (PMC10628438; doi:10.1093/bioinformatics/btad644)
Supplement: btad644_Supplementary_Data [file btad644_supplementary_data.zip › Supplementary file 4.html]

# 


0%
