# Supplementary material for: Inference of differential key regulatory networks and mechanistic drug repurposing candidates from scRNA-seq data with SCANet
Source: Bioinformatics. 2023 Oct 20;39(11):btad644. doi: 10.1093/bioinformatics/btad644 (PMC10628438; doi:10.1093/bioinformatics/btad644)
Supplement: btad644_Supplementary_Data [file btad644_supplementary_data.zip › Supplementary file 2.pdf]

Introduction

As single-cell RNA sequencing technology advances rapidly, it is conceivable to investigate gene connections in a cell type-specific way. However, Single-cell network inference and analysis is a non-trivial task that requires solid statistical background, especially for data preprocessing and proper interpretation of results. As a result, novice researchers frequently struggle to choose the most suitable algorithms for their projects. In addition, several packages are needed for each step of a standard network analysis, and their distinct syntaxes can hinder interoperability between packages, particularly for non-advanced Python users.

Here we propose SCANet, a new python package that incorporates the inference of gene co-expression networks from single-cell gene expression data and a complete analysis of the identified modules through trait and cell type associations, hub genes detection, deciphering of co-regulatory signals in co-expression, and drug-gene interactions identification. This will likely accelerate network analysis pipelines and advance systems biology research.

In this Jupyter Notebook, we explored all aspects of gene coexpression networks (GCNs) using SCANet through a full analysis of the 3k PBMCs from 10x Genomics.

In [65]:
```
# Install SCANet
# https://pypi.org/project/scanet/
# pip install scanet
```

In [66]:
```
# Import modules, packages, functions, etc ...

import scanet as sn
import pandas as pd
```

Read the data

3k PBMCs from 10x Genomics.
The data consists in 3k PBMCs from a Healthy Donor and is freely available from 10x Genomics.
The exact same data is also used in Seurat’s basic clustering tutorial.
Its freely available scanpy.datasets.pbmc3k()

In [67]:
```
pwd
```

Out[67]:
```
'/data/home/baz8031/single-cell/SCANet/scan-final/useage/test/full-example/SCANet-main/docs'
```

In [68]:
```
# Read the data
data_path = "../../data/pbmc3k_raw_.h5ad"
adata = sn.pp.read_h5ad(data_path, pr_process="Yes")
adata
```

```
filtered out 16104 genes that are detected in less than 1 cells
```

Out[68]:
```
AnnData object with n_obs × n_vars = 2700 × 16634
    obs: 'n_genes', 'percent_mito', 'n_counts', 'louvain', 'n_genes_by_counts', 'log1p_n_genes_by_counts',
'total_counts', 'log1p_total_counts', 'total_counts_mt', 'log1p_total_counts_mt', 'pct_counts_mt'
    var: 'gene_ids', 'n_cells', 'mt', 'n_cells_by_counts', 'mean_counts', 'log1p_mean_counts', 'pct_dropou
t_by_counts', 'total_counts', 'log1p_total_counts'
```

In [69]:
```
# Very important the var_names muts be genes names not gene ID
# Checking gene names. Please change if necessary ...!

print(list(adata.var_names)[:15])

# In case not here how to do it
# adata.var = adata.var.set_index('feature_name')
# print(list(adata.var_names)[:15])
```

```
['AL627309.1', 'AP006222.2', 'RP11-206L10.2', 'RP11-206L10.9', 'FAM87B', 'LINC00115', 'FAM41C', 'SAMD11',
'NOC2L', 'KLHL17', 'PLEKHN1', 'RP11-54O7.17', 'HES4', 'RP11-54O7.11', 'ISG15']
```

Step-by-step pr-processing and quality control

Plotting the total number of reads detected per cell.
Cells with few reads are likely to have been broken or failed to capture a cell, and should thus be removed.
Cells with very high reads are likely doublets

In [70]:
```
# Thresholding decision: cells

sn.pp.plot_filter_cells(adata, max_counts=10000, dot=50, fig_size=(12,8))
```

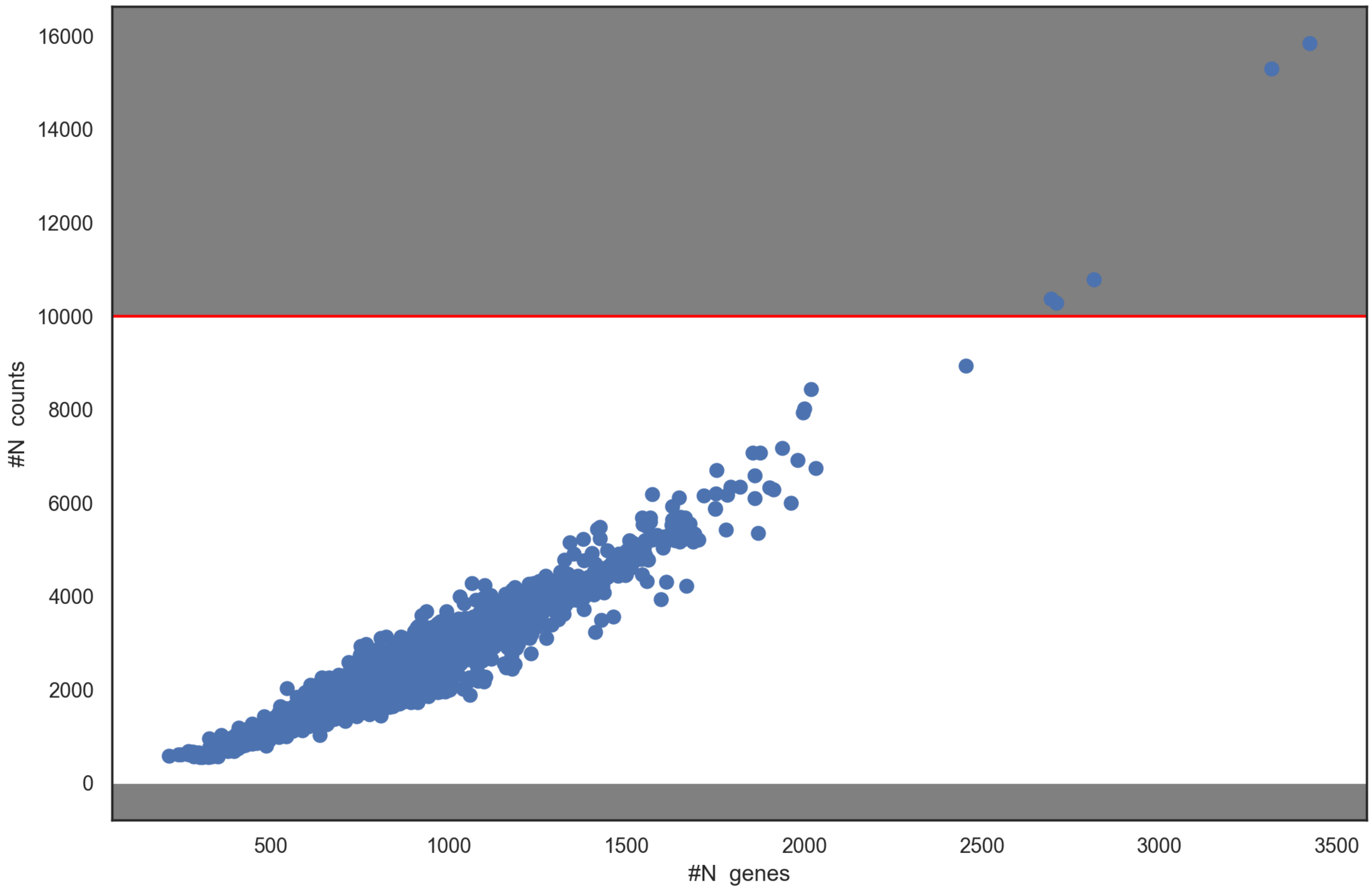


```
Used max_counts is 10000
```

In [71]:
```
# Cells with too many counts are probaly doublet cells/outliers.
# Filtering cells based on #N counts

adata = sn.pp.filter_cells(adata, max_counts=10000)
```

```
Total number of cells: 2700
Number of cells after min genes filter: 2700
filtered out 5 cells that have more than 10000 counts
Number of cells after max count filter: 2695
```

Filter cells based on how many expressed genes (we want to keep it as a normal distribution and avoid noise picks)
In addition to ensuring sufficient sequencing depth for each sample, we also want to make sure that the reads are distributed across the transcriptome. Thus, we count the total number of unique genes detected in each cell.

In [72]:
```
# Thresholding decision: genes
# Use the plot in left to decide on the lowerbound upperbound values

sn.pp.plot_cells_by_n_genes(adata, lowerbound=800, upperbound=800, nbins=100, fig_size=(14,6))
```

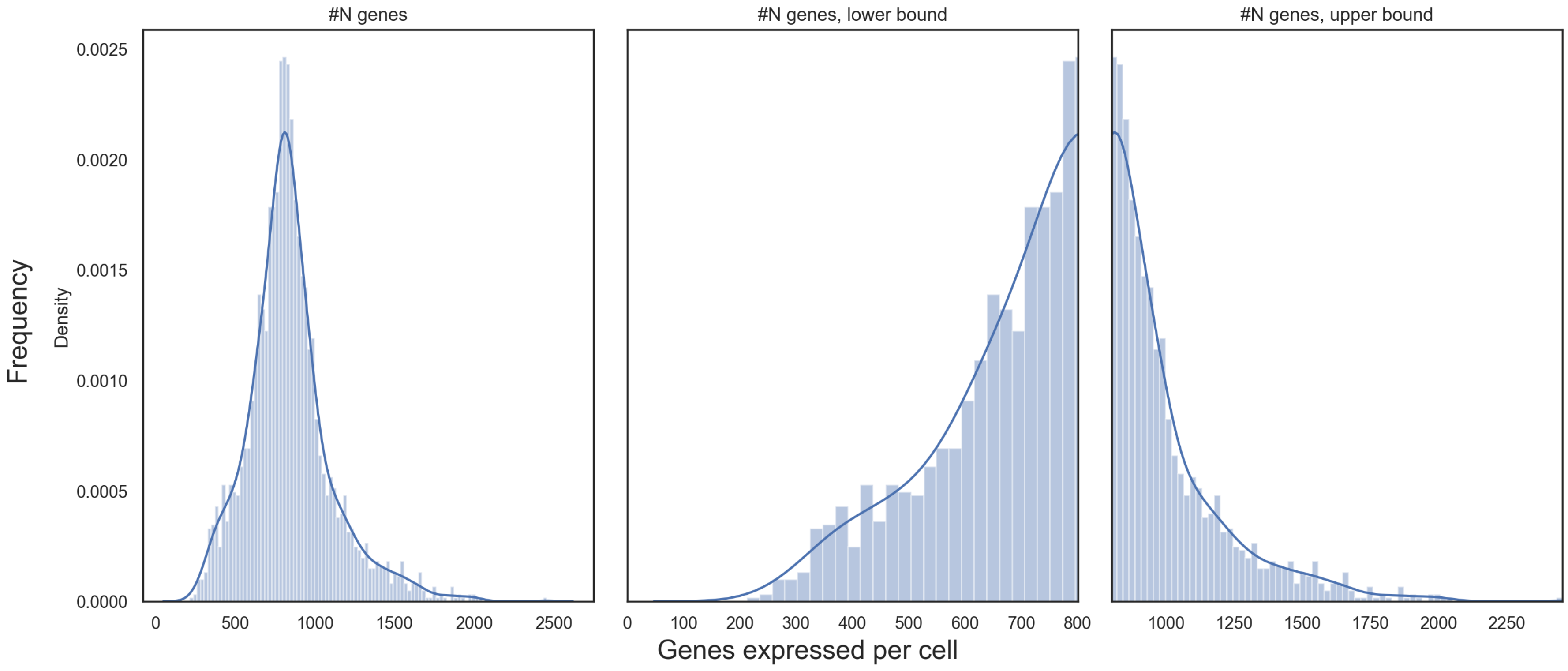

In [73]:
```
#Filtering cells based on #N expressed genes

adata = sn.pp.filter_cells_by_n_genes(adata, min_n_genes=400, max_n_genes=1500)
```

```
Total number of cells: 2695
Number of cells after cell filter: 2522
```

Quality control for genes

It is typically a good idea to remove genes whose expression level is considered "undetectable". We define a gene as detectable if at least two cells contain more than 5 reads from the gene. However, the threshold strongly depends on the sequencing depth. It is important to keep in mind that genes must be filtered after cell filtering since some genes may only be detected in poor quality cells.

In [74]:
```
# Filter (low detected genes) genes based on how mnay cells that they are expressed in

adata = sn.pp.filter_genes(adata, min_cells=3)
```

```
Total number of genes: 16634
filtered out 3188 genes that are detected in less than 3 cells
Number of genes after filtering: 13446
```

Mitochondrial read fractions are only high (light blue color) in particularly low count cells with few detected genes. This could be indicative of damaged/dying cells whose cytoplasmic mRNA has leaked out through a broken membrane, and thus, only mRNA located in the mitochondria is still conserved. These cells are filtered out by our count and gene number thresholds.

In [75]:
```
# Use Mitochondrial gene expression levels to filter out broken cells.

sn.pp.plot_filter_mitochondrial(adata, thres=5, dot=50, fig_size=(8,6))
adata = sn.pp.filter_mitochondrial(adata, max_mt_percent=5)
```

```
Total number of cells: 2522
Number of cells after MT filter: 2481
```

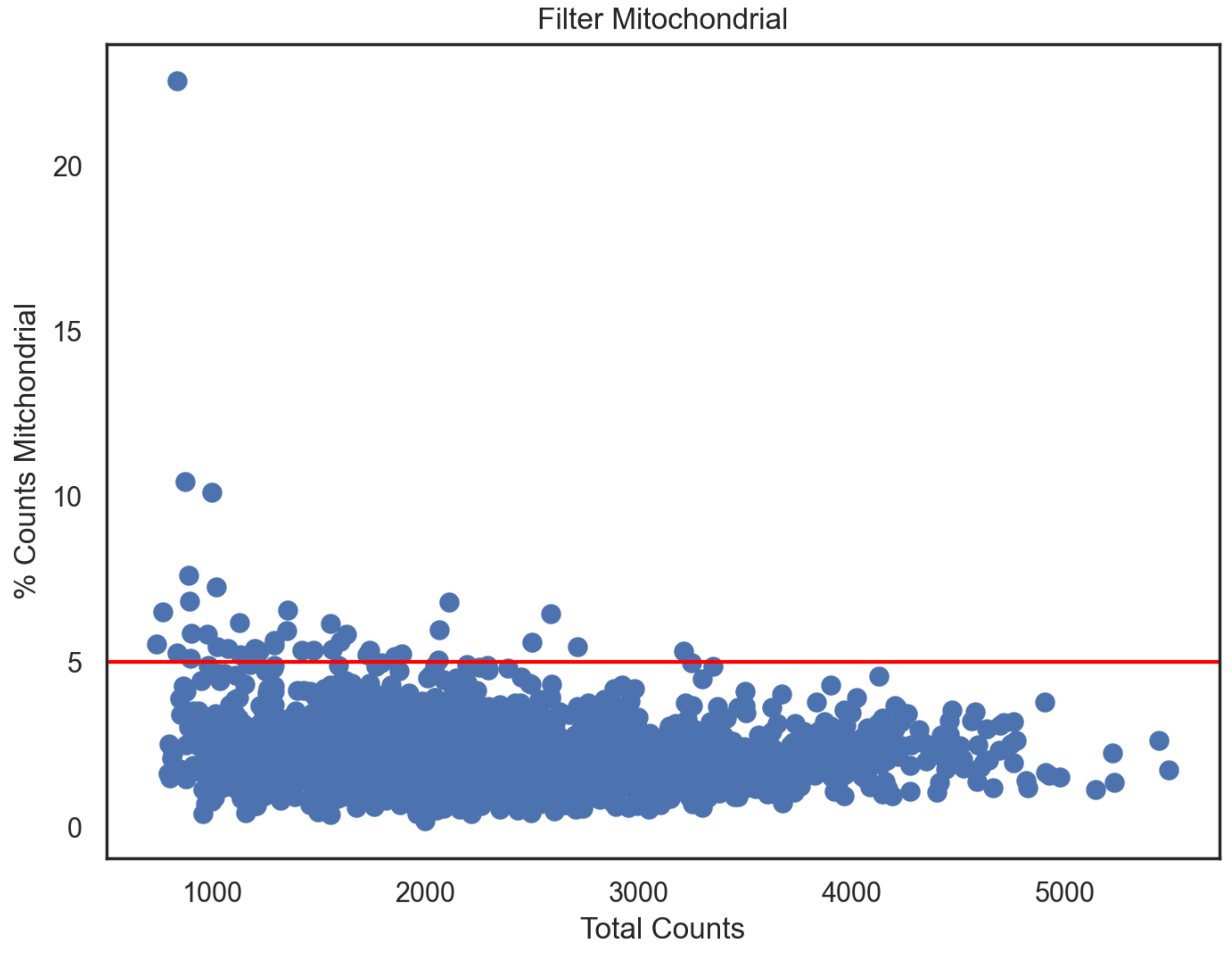

In [76]:
```
# Total-count normalize (library-size correct)
# The data matrix X to 10,000 reads per cell, so that counts become comparable among cells.

adata = sn.pp.log_normalize(adata, target_sum=1e6, use_log=True)
```

```
normalizing counts per cell
    finished (0:00:00)
```

Highly variable gene.

Highly variable gene discovery allows the detection of genes that contribute strongly to cell-to-cell variation within a homogeneous cell population.

In [77]:
```python
# Use only highly variable genes for the analysis not all genes !!!

adata = sn.pp.extract_highly_variable_genes(adata, n_top_genes=2000, plot=True)
adata = adata[:, adata.var.highly_variable]
```

```
If you pass `n_top_genes`, all cutoffs are ignored.
extracting highly variable genes
    finished (0:00:00)
--> added
    'highly_variable', boolean vector (adata.var)
    'means', float vector (adata.var)
    'dispersions', float vector (adata.var)
    'dispersions_norm', float vector (adata.var)

 Number of highly variable genes: 2000
```

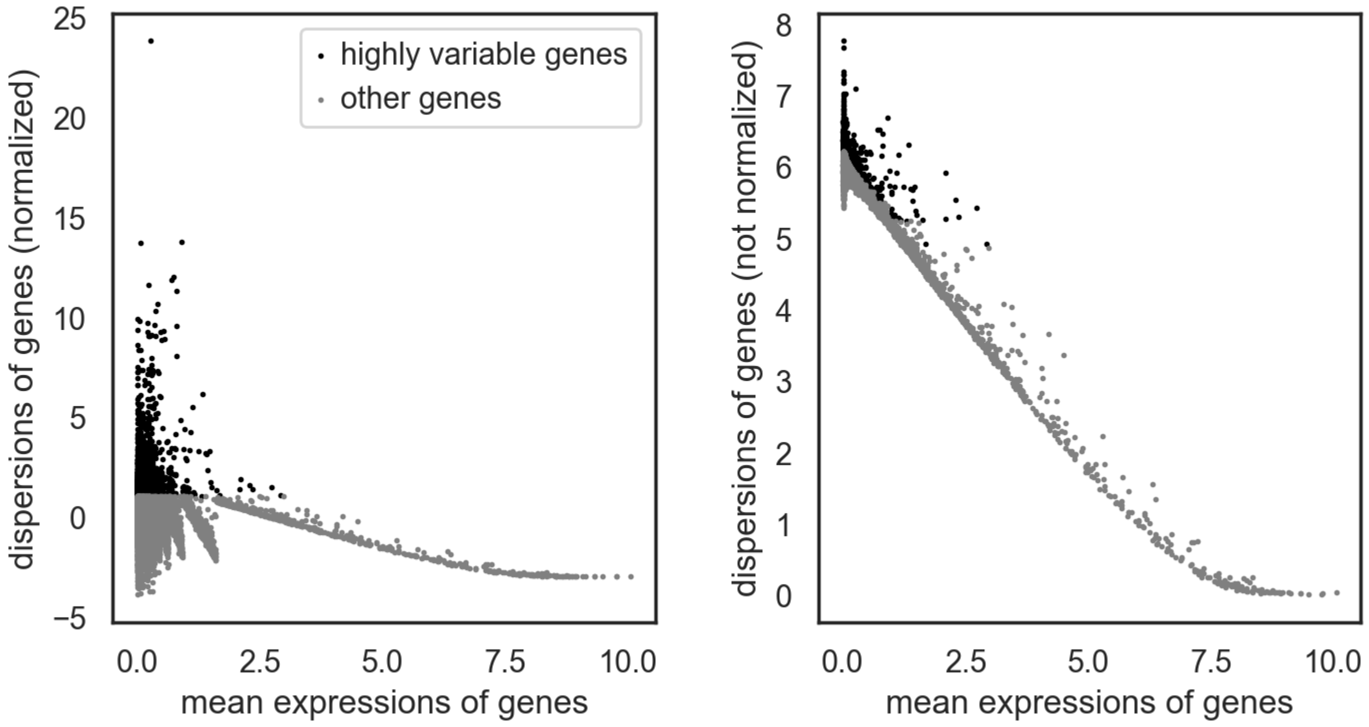

In [78]:
```python
# Let us inspect the contribution of single PCs to the total variance in the data.
# This gives us information about how many PCs we should consider in order to compute
# the neighborhood relations of cells

sn.vz.dimensionality_reduction_parameters(adata)
```

```
computing PCA
    on highly variable genes
    with n_comps=50
    finished (0:00:00)
```

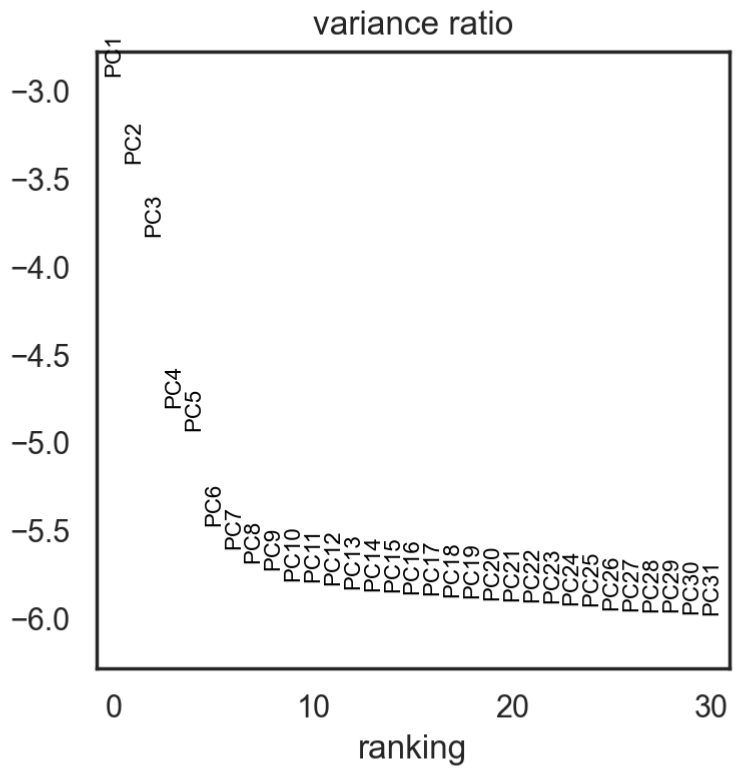

In [114]:
```
# Dimensionality reduction

adata = sn.vz.dimensionality_reduction(adata, method="UMAP", n_pcs=12, n_neighbors=10)
```

```
Calculating dimensionality reduction :UMAP
computing neighbors
    using 'X_pca' with n_pcs = 12
    finished: added to `.uns['neighbors']`
    `.obsp['distances']`, distances for each pair of neighbors
    `.obsp['connectivities']`, weighted adjacency matrix (0:00:00)
computing UMAP
    finished: added
    'X_umap', UMAP coordinates (adata.obsm) (0:00:00)
it tooks :0.016199008623758 95 minutes.
```

In [115]:
```
# Seclect the annotation you want to work with:
# Available annotations:"
print(list(adata.obs))
```

```
['n_genes', 'percent_mito', 'n_counts', 'louvain', 'n_genes_by_counts', 'log1p_n_genes_by_counts', 'total_
counts', 'log1p_total_counts', 'total_counts_mt', 'log1p_total_counts_mt', 'pct_counts_mt']
```

In [116]:
```
cell_annoatation="louvain"
```

In [117]:
```
# Visualization

cell_annoatation="louvain"
sn.vz.visualization(adata, method="UMAP", color=cell_annoatation)
```

```
 Available method are: PCA, t-SNE,  UMAP, or ALL
```

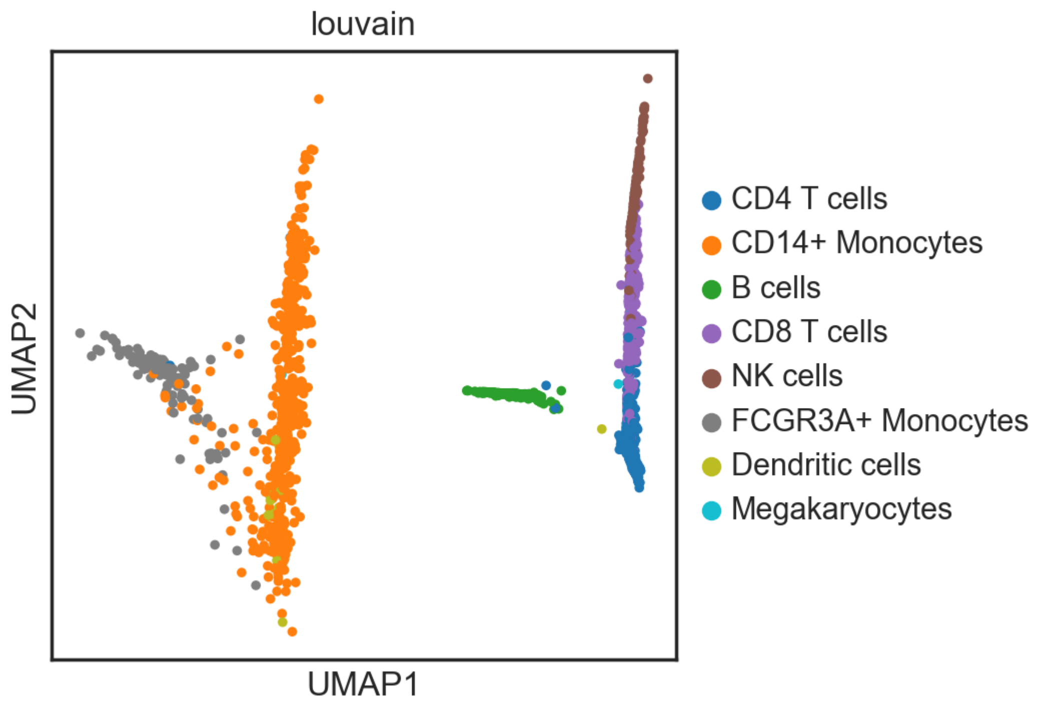

In [118]:
```
# Saving the data

name_reduced = "data/"+data_path.split("/")[-1].split(".h5ad")[0]+"_processed.h5ad"
print(name_reduced)
adata.write_h5ad(name_reduced)
```

```
data/pbmc3k_raw__processed.h5ad
```

In [119]:
```
adata
```

Out[119]:
```
AnnData object with n_obs × n_vars = 2481 × 2000
    obs: 'n_genes', 'percent_mito', 'n_counts', 'louvain', 'n_genes_by_counts', 'log1p_n_genes_by_counts',
'total_counts', 'log1p_total_counts', 'total_counts_mt', 'log1p_total_counts_mt', 'pct_counts_mt'
    var: 'gene_ids', 'n_cells', 'mt', 'n_cells_by_counts', 'mean_counts', 'log1p_mean_counts', 'pct_dropou
t_by_counts', 'total_counts', 'log1p_total_counts', 'highly_variable', 'means', 'dispersions', 'dispersion
s_norm'
    uns: 'log1p', 'hvg', 'pca', 'neighbors', 'umap', 'louvain_colors'
    obsm: 'X_pca', 'X_umap'
    varm: 'PCs'
    obsp: 'distances', 'connectivities'
```

Representative cells

Grouping of neighboring single cells where the data from every single cell is combined into a single pseudo-sample that represents the overall signal across these cells. Cells for each cell type are sub-clustered into N (num_rep_cells) sub-cluster each sub-cluster is then turned into a single representative cell by averaging the gene expression profiles across its cells.

In [120]:
```
# Representative cells

adata_r = sn.pb.representative_cells(adata, num_rep_cells=50, cell_anno=cell_annoatation)
```

```
clusters in the data louvain
CD4 T cells          1110
CD14+ Monocytes       448
B cells               319
CD8 T cells           307
NK cells              151
FCGR3A+ Monocytes     124
Dendritic cells        18
Megakaryocytes          4
dtype: int64

The following cell clusters will be deletedas their size if less than the specified number ofrepresentativ
e cells:
['Dendritic cells', 'Megakaryocytes']
data type: SparseCSRView

Selected representative cells for each cluster __SCANclusters__
B cells              50
CD14+ Monocytes      50
CD4 T cells          50
CD8 T cells          50
FCGR3A+ Monocytes    50
NK cells             50
dtype: int64
```

In [121]:
```
# The reduced data

adata_r
```

Out[121]:
```
AnnData object with n_obs × n_vars = 300 × 2000
    obs: '__SCANclusters__'
    var: 'Genes'
```

In [122]:
```
sn.vz.dimensionality_reduction_parameters(adata_r)
```

```
computing PCA
    with n_comps=50
    finished (0:00:02)
```

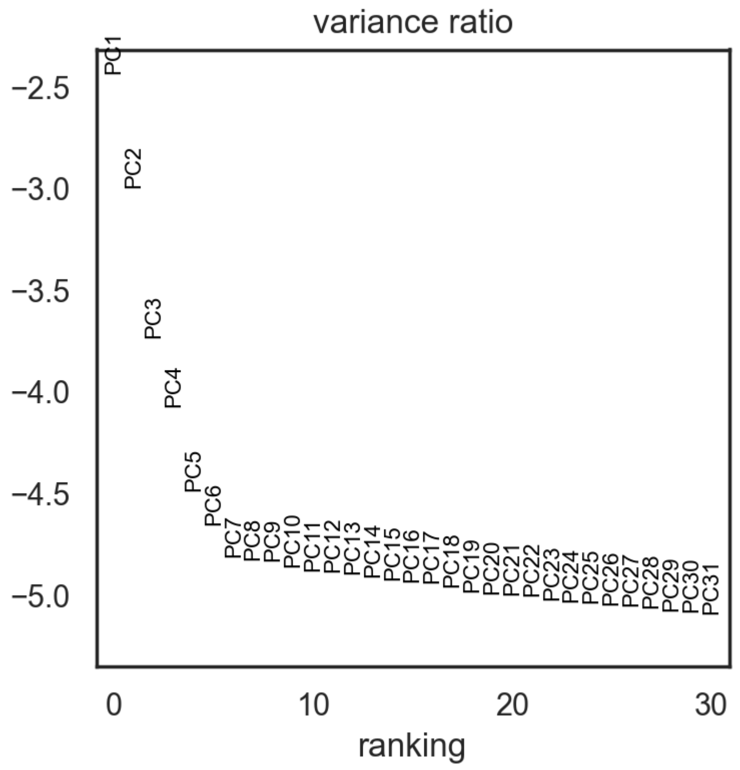

In [123]:
```
adata_new = sn.vz.dimensionality_reduction(adata_r, method="UMAP", n_pcs=10, n_neighbors=5)
```

```
Calculating dimensionality reduction :UMAP
computing neighbors
    using 'X_pca' with n_pcs = 10
    finished: added to `.uns['neighbors']`
    `.obsp['distances']`, distances for each pair of neighbors
    `.obsp['connectivities']`, weighted adjacency matrix (0:00:00)
computing UMAP
    finished: added
    'X_umap', UMAP coordinates (adata.obsm) (0:00:00)
it tooks :0.0017033775647481283 minutes.
```

In [124]:
```
sn.vz.visualization(adata_new, method="UMAP", color='__SCANclusters__')
```

```
... storing '__SCANclusters__' as categorical

 Available method are: PCA, t-SNE,  UMAP, or ALL
```

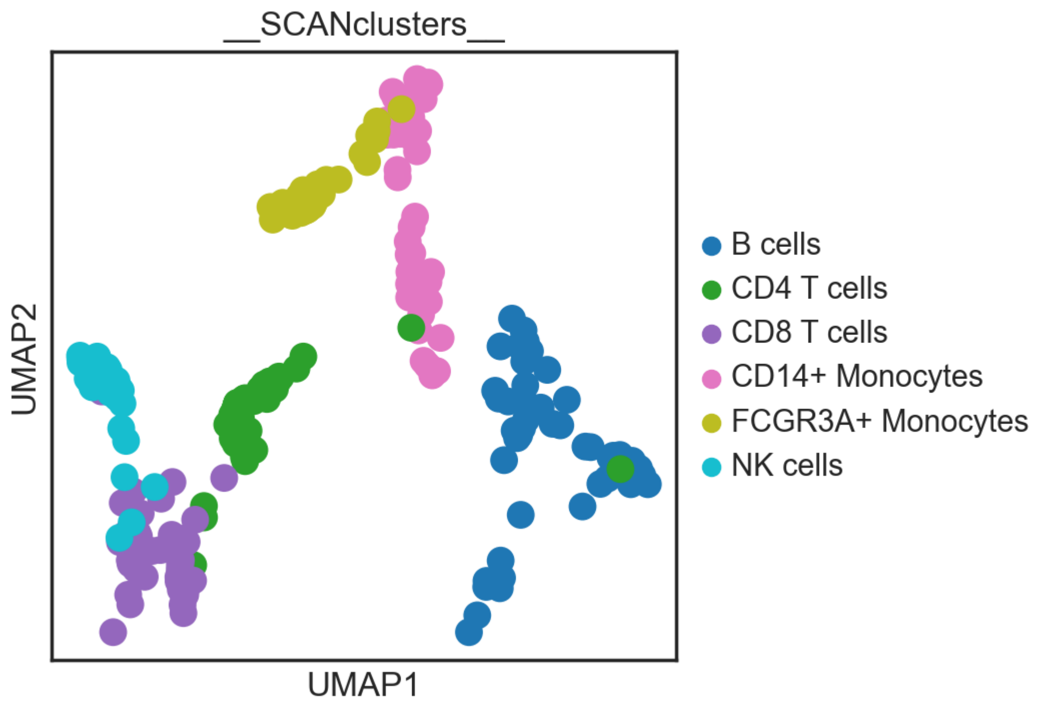

In [125]:
```
# Saving the data

name_reduced = "data/"+data_path.split("/")[-1].split(".h5ad")[0]+"_reduced.h5ad"
print(name_reduced)
adata_r.write_h5ad(name_reduced)
```

```
data/pbmc3k_raw__reduced.h5ad
```

CO-expression analysis

In [127]:
```
# Read the reduced data

data_path = "data/pbmc3k_raw__reduced.h5ad"
adata_r = sn.pp.read_h5ad(data_path)
adata_r
```

Out[127]:
```
AnnData object with n_obs × n_vars = 300 × 2000
    obs: '__SCANclusters__'
    var: 'Genes'
    uns: '__SCANclusters___colors', 'neighbors', 'pca', 'umap'
    obsm: 'X_pca', 'X_umap'
    varm: 'PCs'
    obsp: 'connectivities', 'distances'
```

we can reconstruct a gene coexpression network (GCN) with the WGCNA algorithm (Langfelder and Horvath 2008). First of all, we need to identify the most suitable SFTpower power that makes the network satisfy the scale-free topology. We do that with the function plot_powers(). Correlation values are raised to a power SFTpower to amplify their distances and, hence, to make the module detection algorithm more powerful. The higher the value of SFTpower, the closer to the scale-free topology the network is. However, a very high SFTpower power reduces mean connectivity, which is not desired. To solve this trade-off, we pick the lowest SFTpower power above a certain threshold 0.8. This makes the network close to the scale-free topology without dramatically reducing the mean connectivity.

Possible parameter values

net_type Network type. One of ’signed’, ’signed hybrid’ or ’unsigned’. Default: ’signed’.
module_merging_threshold Correlation threshold to merge similar modules into a single one. Default: 0.8.
SFTpower SFT power generated by the function plot_powers.
cor_method Correlation method. One of "pearson", "biweight" or "spearman". Default is "spearman".

In [128]:
```
# Suitable SFTpower power that makes the network satisfy the scale-free topology

SFTpower = sn.co.plot_powers(adata_r, network_type="unsigned", cor_method="pearson")
SFTpower
```

```
Data dimension is : 300 Cells and 2000 Genes
[1] 2000  300
[1] "Removing outlier cells ..."
[1] "Removed 1 cells ..."
[1] 2000  299
```

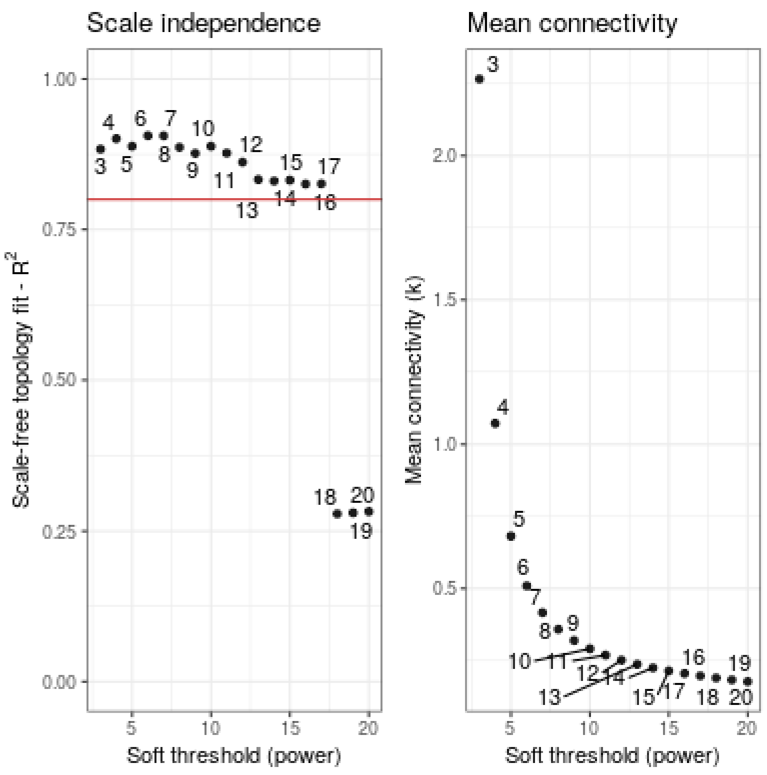


```
The optimal power to use is : 3.0 or visualize the plot and change the value accordingly.
Tip: If power values are not exponential distributed dont use the maximum value.
```

Out[128]: 3

In [129]:
```
# Infer the GCN

net = sn.co.co_expression(adata_r, network_type="unsigned", cor_method="pearson",
                          power=SFTpower, module_merging_threshold=0.8)
```

```
Data dimension is : 300 Cells and 2000 Genes
[1] 2000  300
[1] "Removing outlier cells ..."
[1] "Removed 1 cells ..."
[1] 2000  299
..connectivity..
..matrix multiplication (system BLAS)..
..normalization..
..done.
```

In [130]:
```python
# Dendrogram of genes and modules

sn.co.plot_dendrogram(net,  fig_size=(800,400))
```

```
Plotting the dendrogram
NULL
```

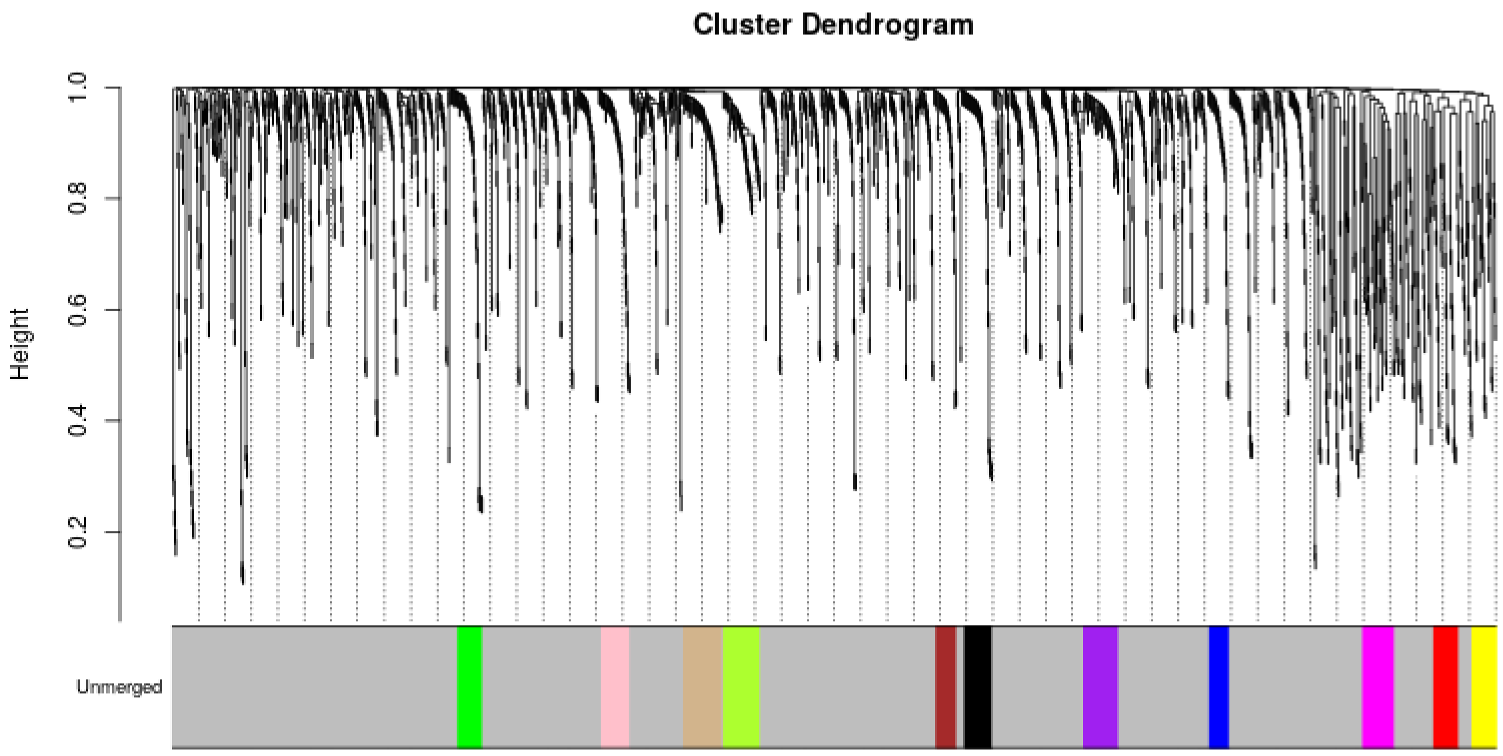

In [131]:
```python
# Heatmap of pairwise correlations between module eigengenes

sn.co.plot_eigengene_network(net, fig_size=(800,400))
```

```
Plotting the eigengene network
NULL
```

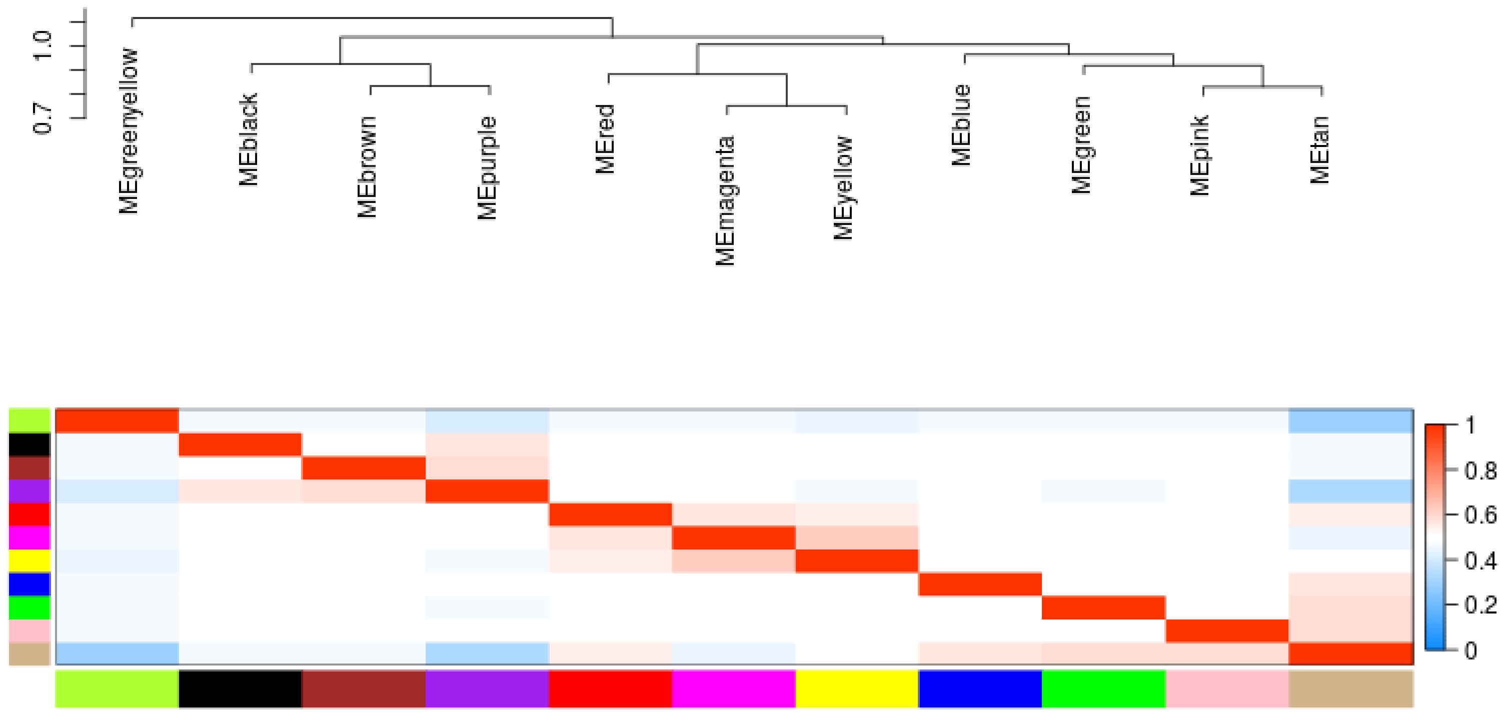

In [132]:
```python
# Number of genes per module.

df_modules = sn.co.plot_modules(net, figsize=(8,4))
df_modules.head()
```

```
Plotting the modules
```

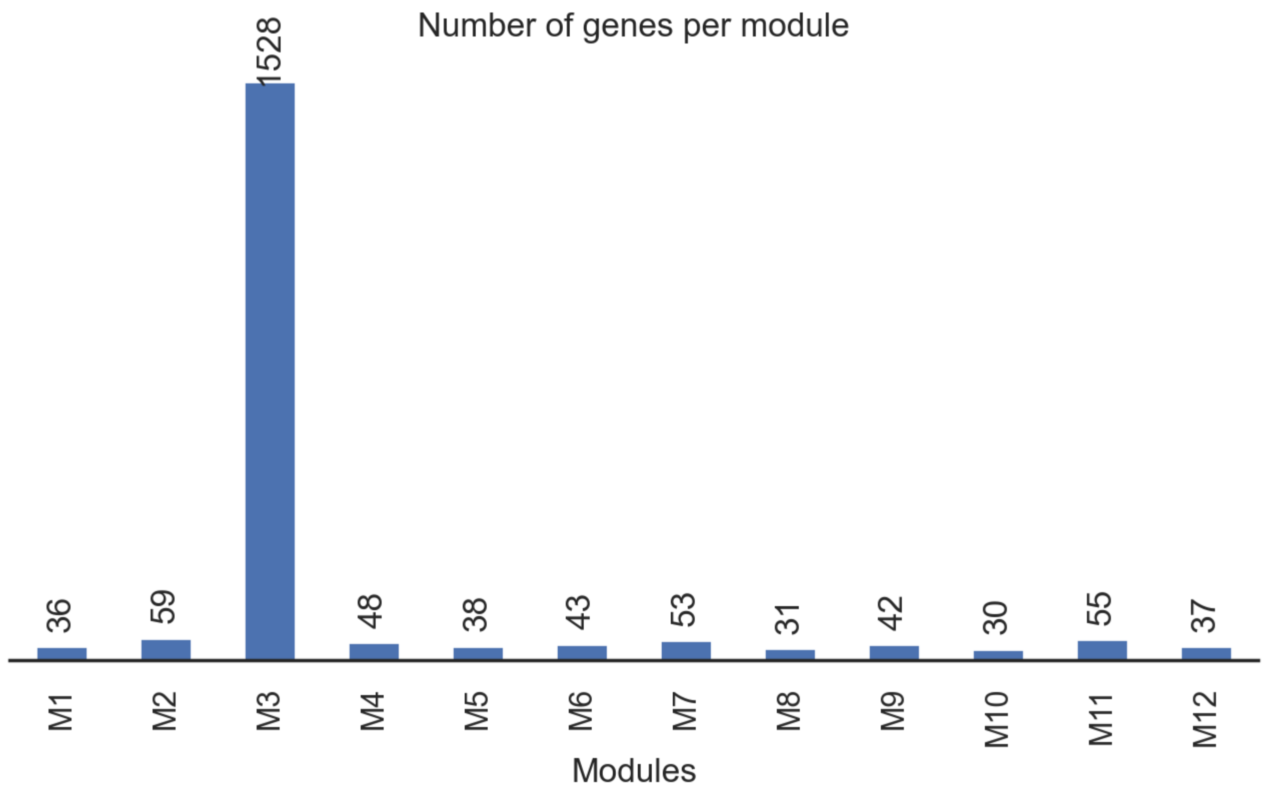

Out[132]:

	Module_r	n_genes	genes	Modules
**0**	yellow	36	["AIM1L", "SSX2IP", "RP11-134G8.7", "SOX13", "...	M1
**1**	tan	59	["STMN1", "RHOC", "CD160", "SH2D2A", "FCRL6", ...	M2
**2**	grey	1528	["TNFRSF4", "ATAD3C", "MMP23B", "CDK11B", "SLC...	M3
**3**	magenta	48	["CA6", "RP3-329E20.2", "PIFO", "AQP10", "SLC1...	M4
**4**	red	38	["SLC2A5", "SLC2A1-AS1", "C1orf177", "RP11-356...	M5

Hub gene identification

Hub genes are often identified using two different metrics: module membership (MM) (i.e., correlation of a gene to its module eigengene) and degree (i.e., sum of connection weights of a gene to all other genes in the module). Some researchers consider the top 10% genes with the highest degree as hubs, while others consider those with MM > 0.8. To avoid false positives, we combine both metrics and defines hub genes as the top 10% genes with highest degree that have MM > 0.8. Hubs can be identified with the function hub_genes().

In [133]:
```
# Hub genes

hub_genes_df = sn.co.hub_genes(adata=adata_r, net=net, figsize=(6,4))
hub_genes_df.head(5)
```

```
Data dimension is : 300 Cells and 2000 Genes
[1] 2000  300
[1] "Removing outlier cells ..."
[1] "Removed 1 cells ..."
[1] 2000  299
```

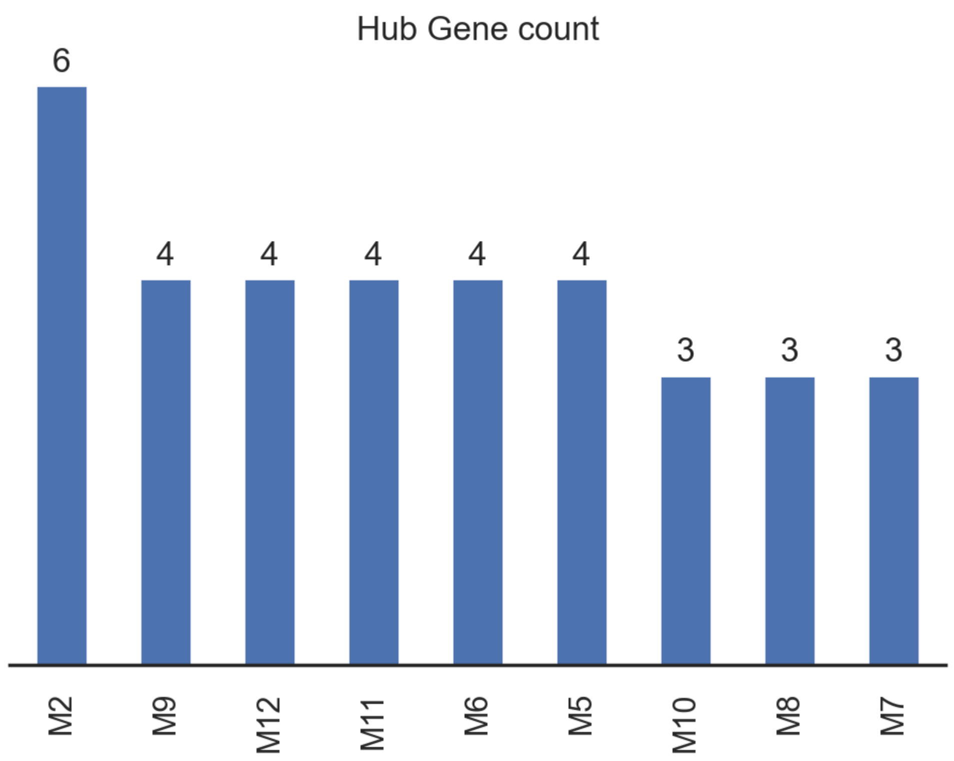

Out[133]:

	Gene	kWithin	Modules
**0**	RP11-277P12.20	7.392159	M9
**1**	WDR66	7.358127	M9
**2**	CTB-113I20.2	7.273552	M9
**3**	MEIG1	7.167886	M9
**4**	RP11-96K19.2	5.477152	M10

Module-Annotation associations

The function modules_to_annotation_cor() can be used to calculate Module-Annotation correlations. This analysis is useful to identify modules that are positively or negatively correlated with particular annotation (such as cell type or diseased viruses healthy), which means that their gene expression levels go up or down in these conditions. Here, cell type will be considered, so we want to identify groups of genes whose expression levels are inhibited or induced in particular cell type.

In [134]:
```
cor = sn.co.modules_to_annotation_cor(adata_r, net, figsize=(12,6), cor_method="spearman")
cor.head(5)
```

```
Data dimension is : 300 Cells and 2000 Genes
[1] 2000  300
[1] "Removing outlier cells ..."
[1] "Removed 1 cells ..."
[1] 2000  299
```

Out[134]:

	Modules	annotation	cor	pvalue
1	M9	B.cells	0.374891	2.065767e-11
2	M9	CD14..Monocytes	-0.142679	1.353137e-02
3	M9	CD4.T.cells	-0.028057	6.289442e-01
4	M9	CD8.T.cells	-0.077924	1.790001e-01
5	M9	FCGR3A..Monocytes	-0.167977	3.577883e-03

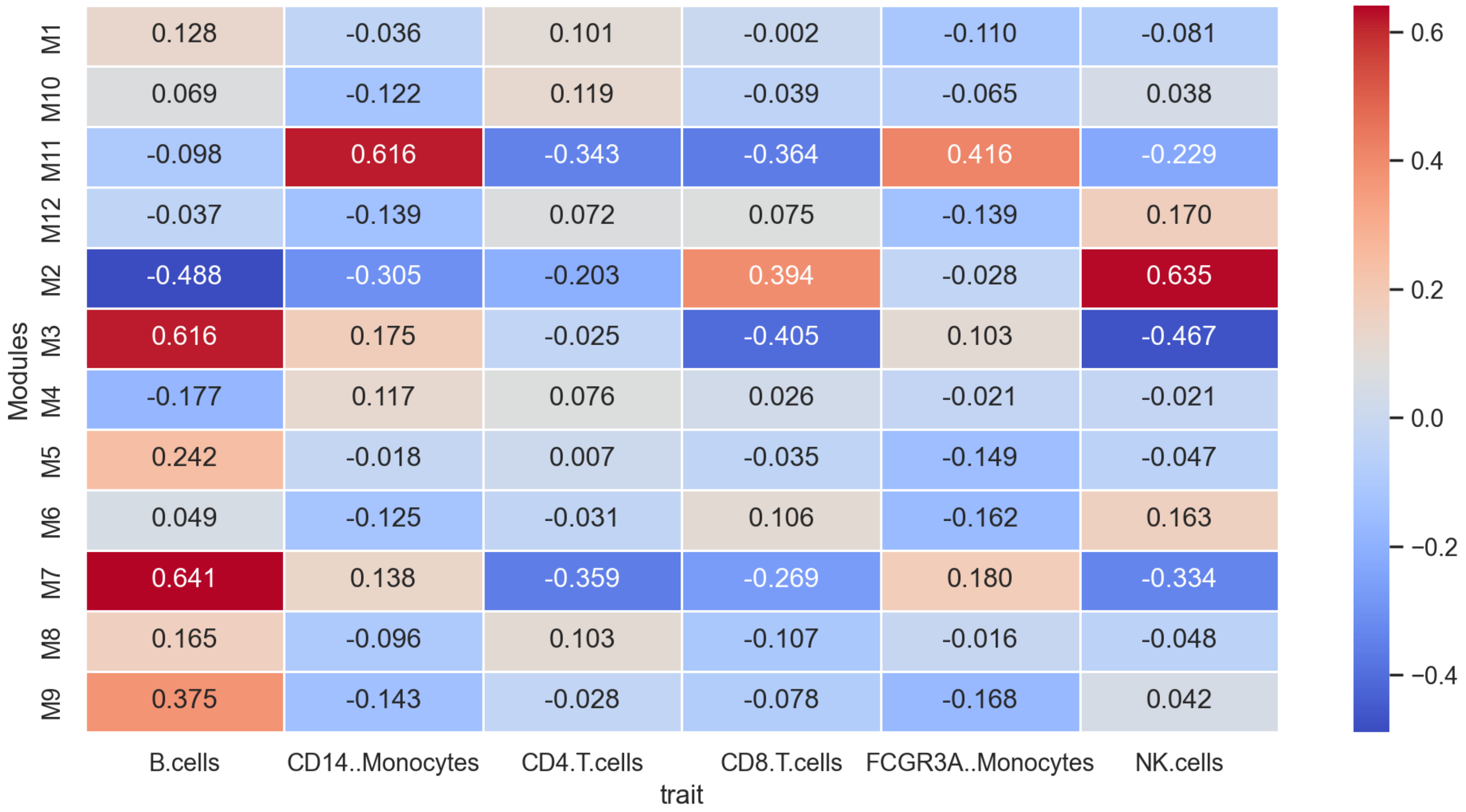

In [135]:
```
# Filter only the modules that are highly correlated (or >= 0.5)

cor__ = cor[abs(cor["cor"])>=0.5]
cor__
```

Out[135]:

	Modules	annotation	cor	pvalue
26	M11	CD14..Monocytes	0.615684	1.382677e-32
31	M3	B.cells	0.615836	1.321597e-32
49	M7	B.cells	0.641414	4.709380e-36
66	M2	NK.cells	0.635498	3.157962e-35

Analysis of co-expression modules

In [160]:
```
# Select module of interest
# Annotation correlating with the module

M = "M11"
anno_name_ = ["CD14..Monocytes"]
```

In [161]:
```
sn.co.module_to_annotation_cor(cor, module = M, figsize=(8,4))
```

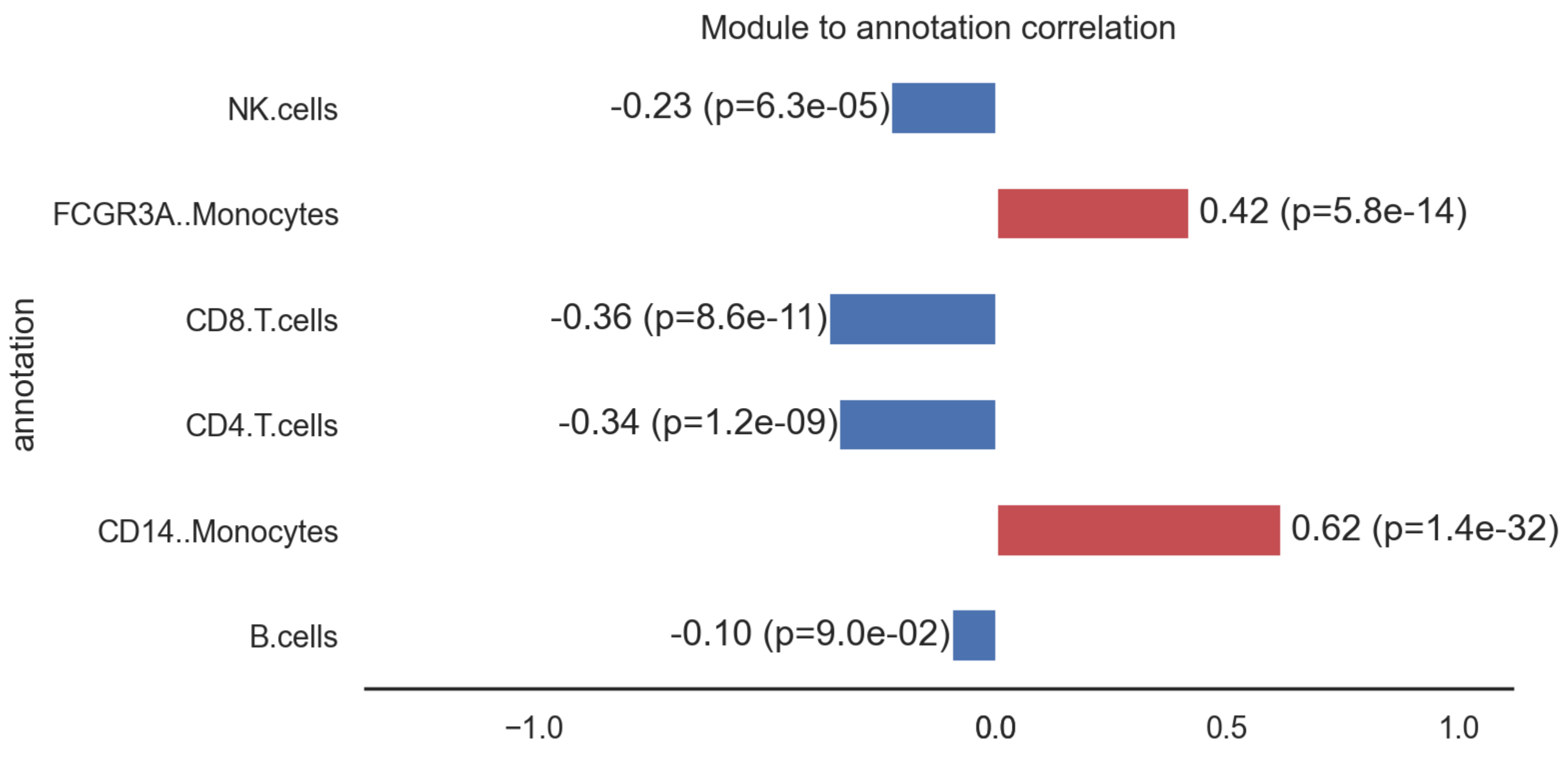

Average module's gene expression in the different cell types

In [162]:
```
sn.co.Module_Genes_Avrage_Expr(module = M, adata = adata_r, figsize=(12,6))
```

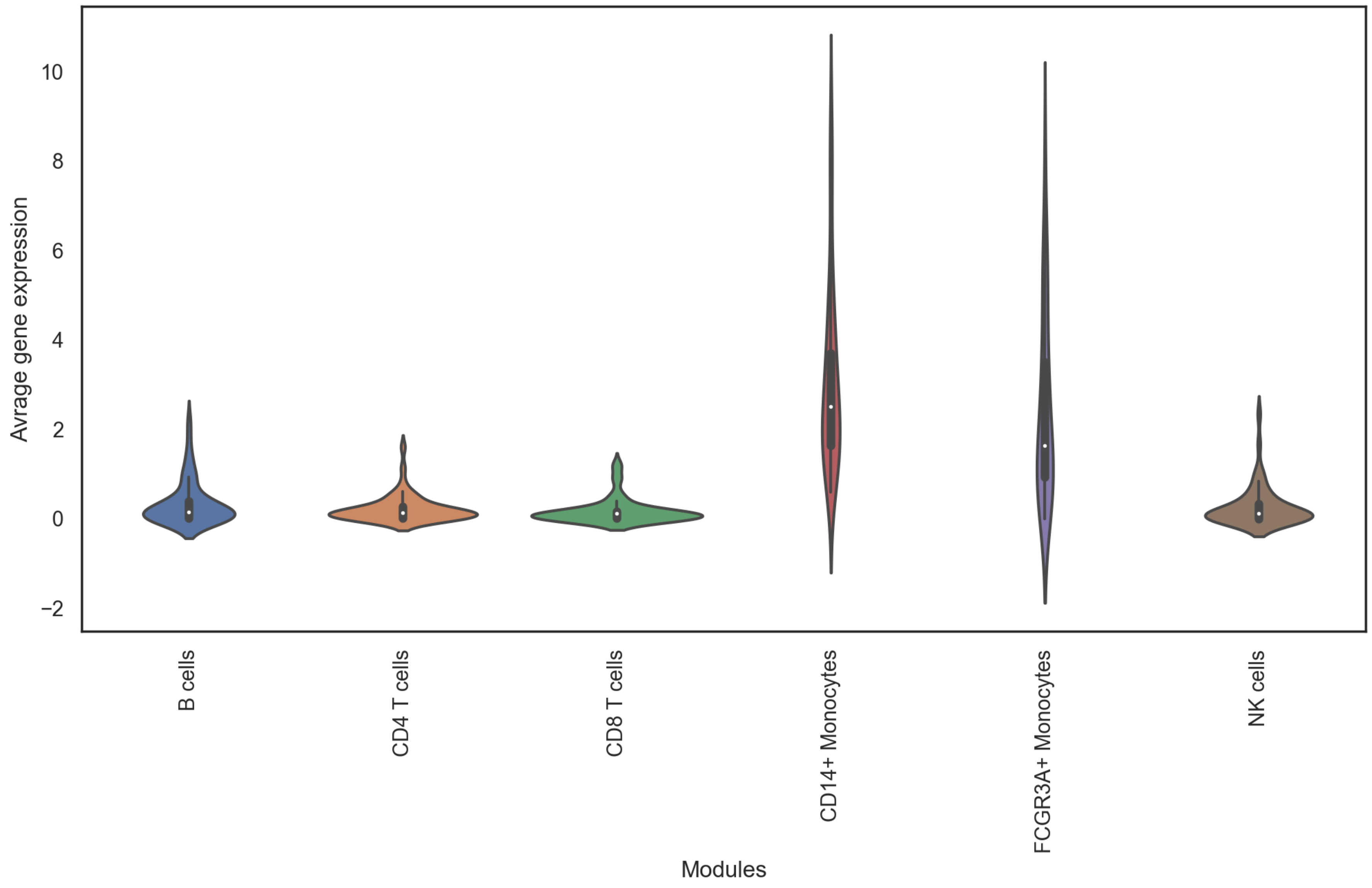

Module membership (MM) (i.e., correlation of a gene to its module eigengene) across different cell types

In [163]:
```
module_membership_df = sn.co.plot_module_membership(net, adata=adata_r, figsize=(20,20))
```

```
Plotting Module Membership
```

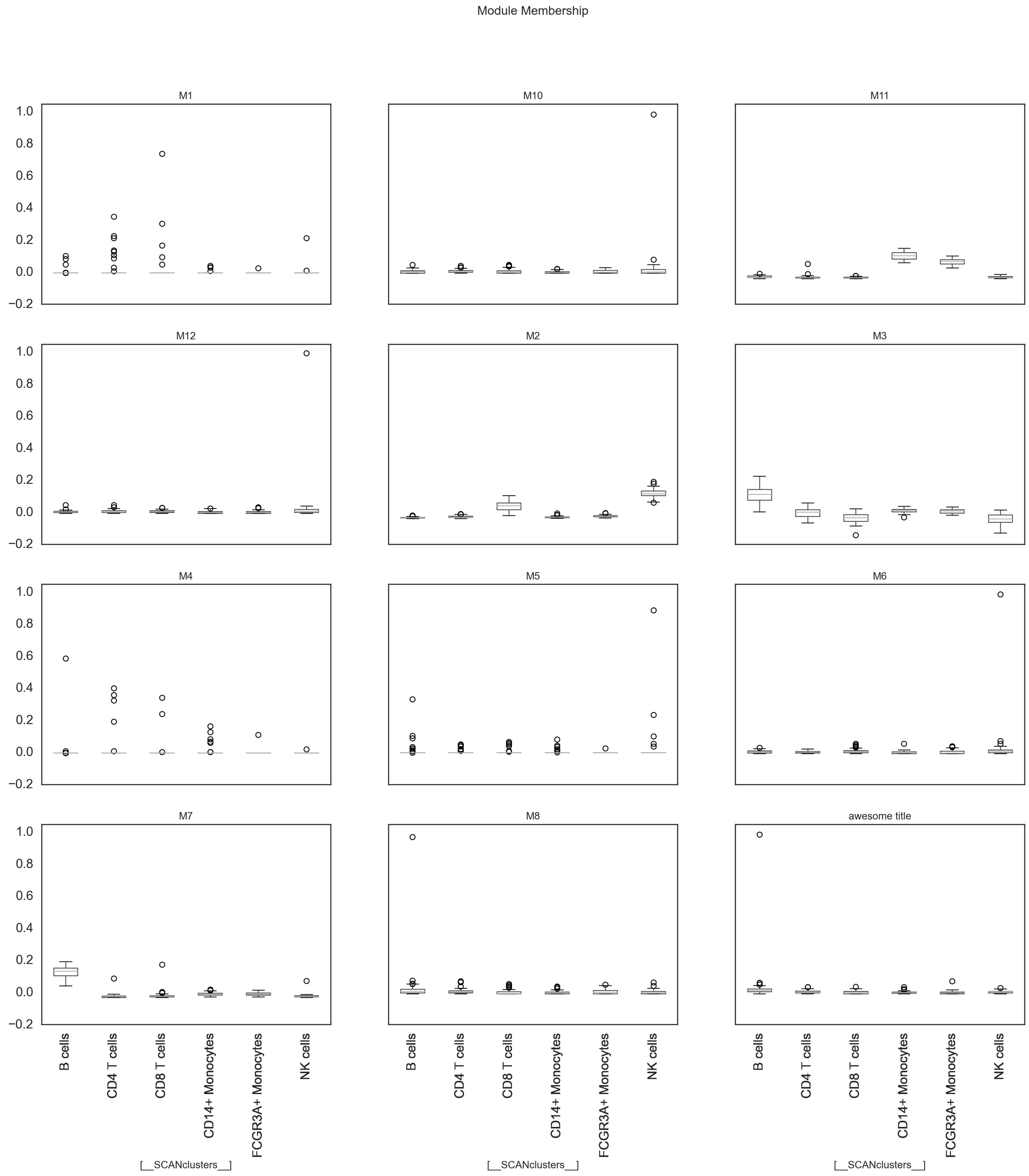

In [164]:
```
# Module membership of the selected module

sn.co.Module_Activity(module=M, plot_type="box", df = module_membership_df, figsize=(12,6))
```

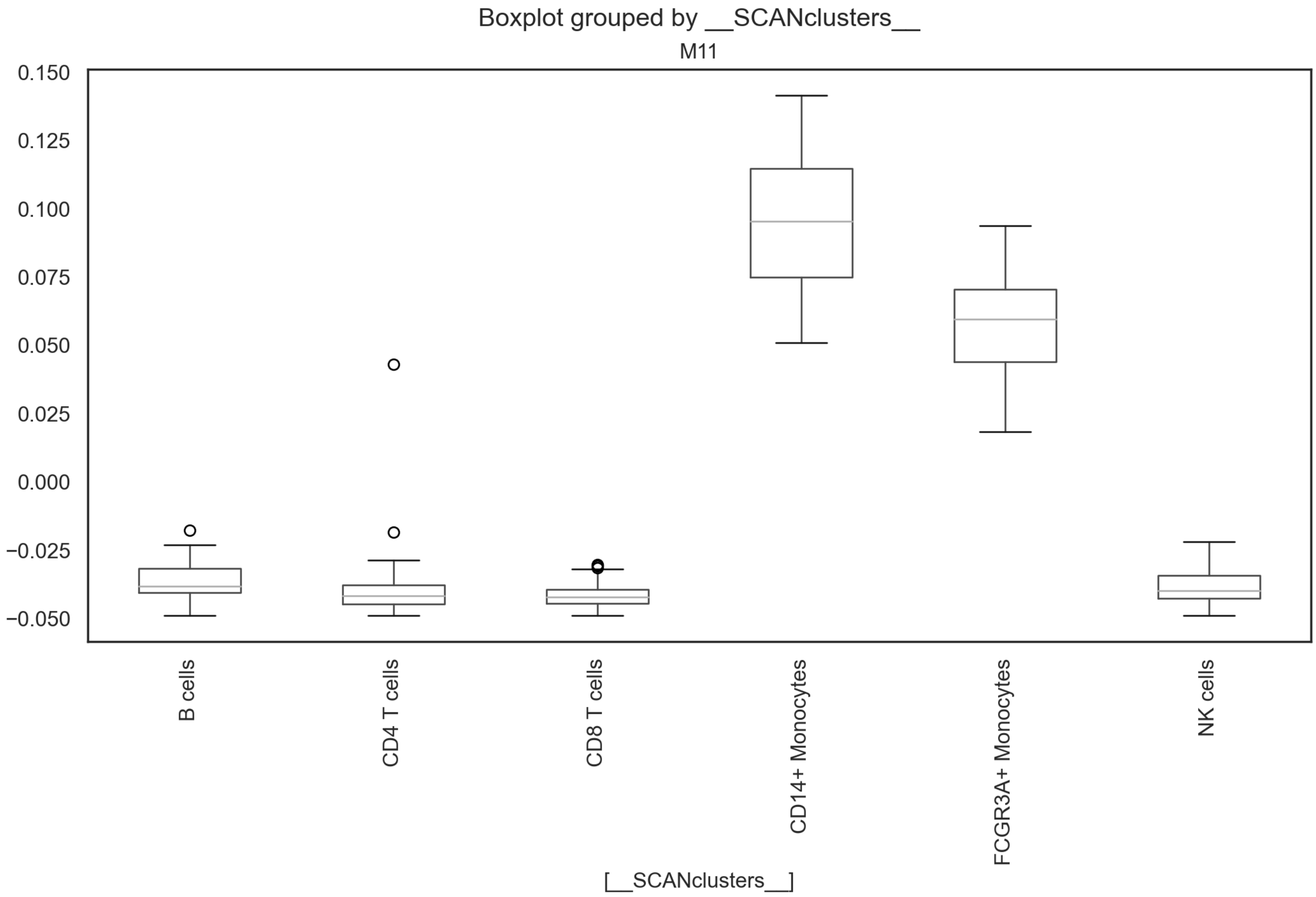

In [165]:
```
# Module membership of the selected module. violin plot.

sn.co.Module_Activity(module=M, plot_type="violin", df = module_membership_df, figsize=(12,6))
```

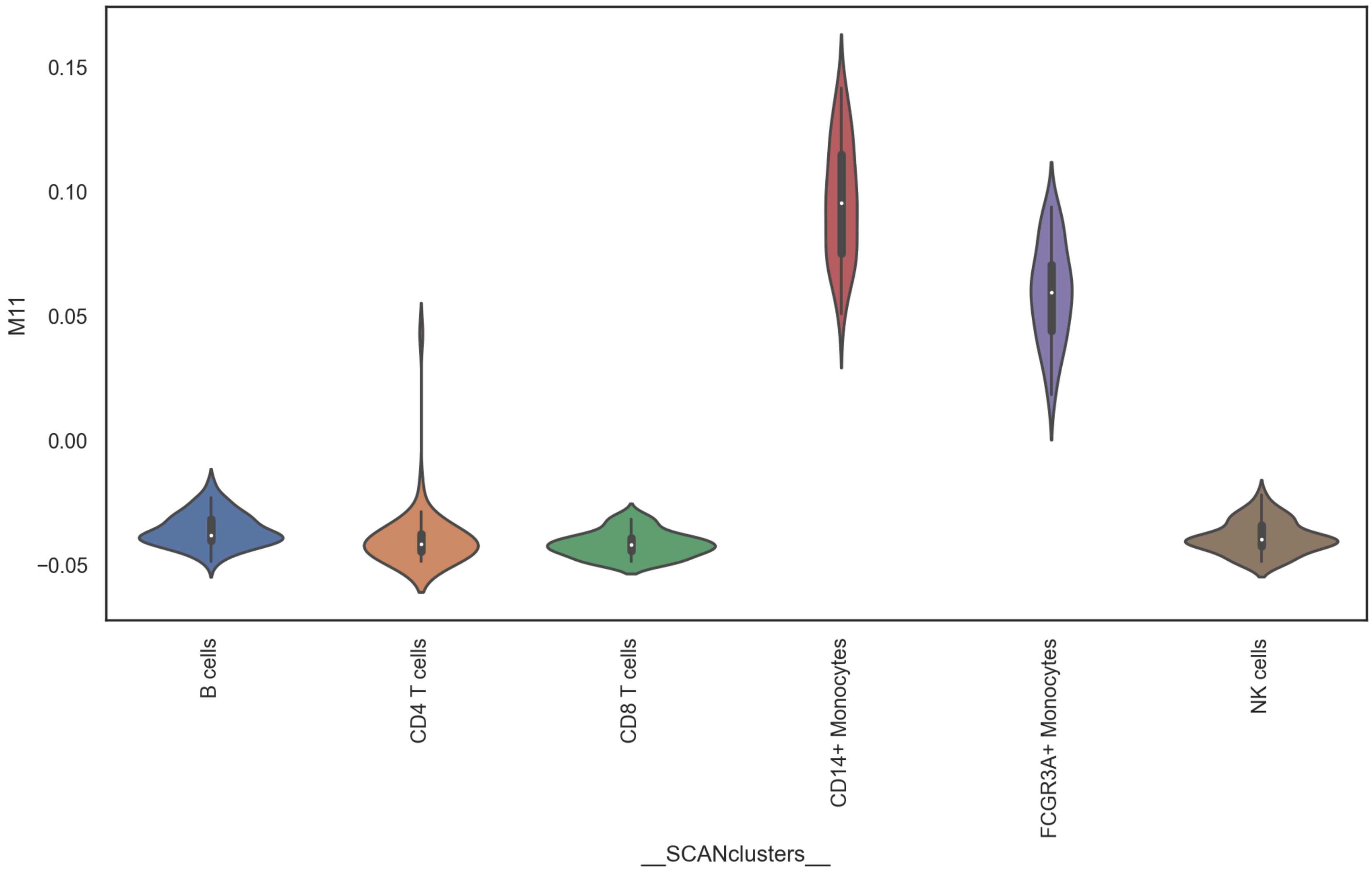

Subgraph extraction can be particularly useful to visualize specific modules, and it can be done with the function get_edge_list(). The function returns the subgraph as an edge list. in order to get the subgraph edges are removed based on minimum correlation (co_cutoff).

In [166]:
```
network_ = sn.co.module_to_network(net, module = M, co_cutoff=0.6)
print(network_.shape)
network_.head(5)
```

```
Your graph fits the scale-free topology. P-value:0.996468420969116
Using a correlation cut-off of 0.6 found 47 edges
There are 17 unique genes
(47, 4)
```

Out[166]:

	Gene1	Gene2	Weight	Module
**279**	S100A9	S100A8	0.743428	M11
**607**	CSF3R	CD14	0.627432	M11
**609**	S100A9	CD14	0.659982	M11
**611**	S100A8	CD14	0.681525	M11
**994**	S100A9	FCN1	0.671737	M11

Network visualization

As we now have an edge list for a module, let's visualize it with the function plot_gcn().
Hub genes and thier edges are given in red

In [167]:
```
sn.pl.plot_gcn(network_=network_,hub_genes_df=hub_genes_df, name="gcn_network", drug_interaction=False,
               smooth_edges=True)
```

```
Hub genes: ['CFD', 'CST3', 'S100A9', 'FCN1']
```

Out[167]:

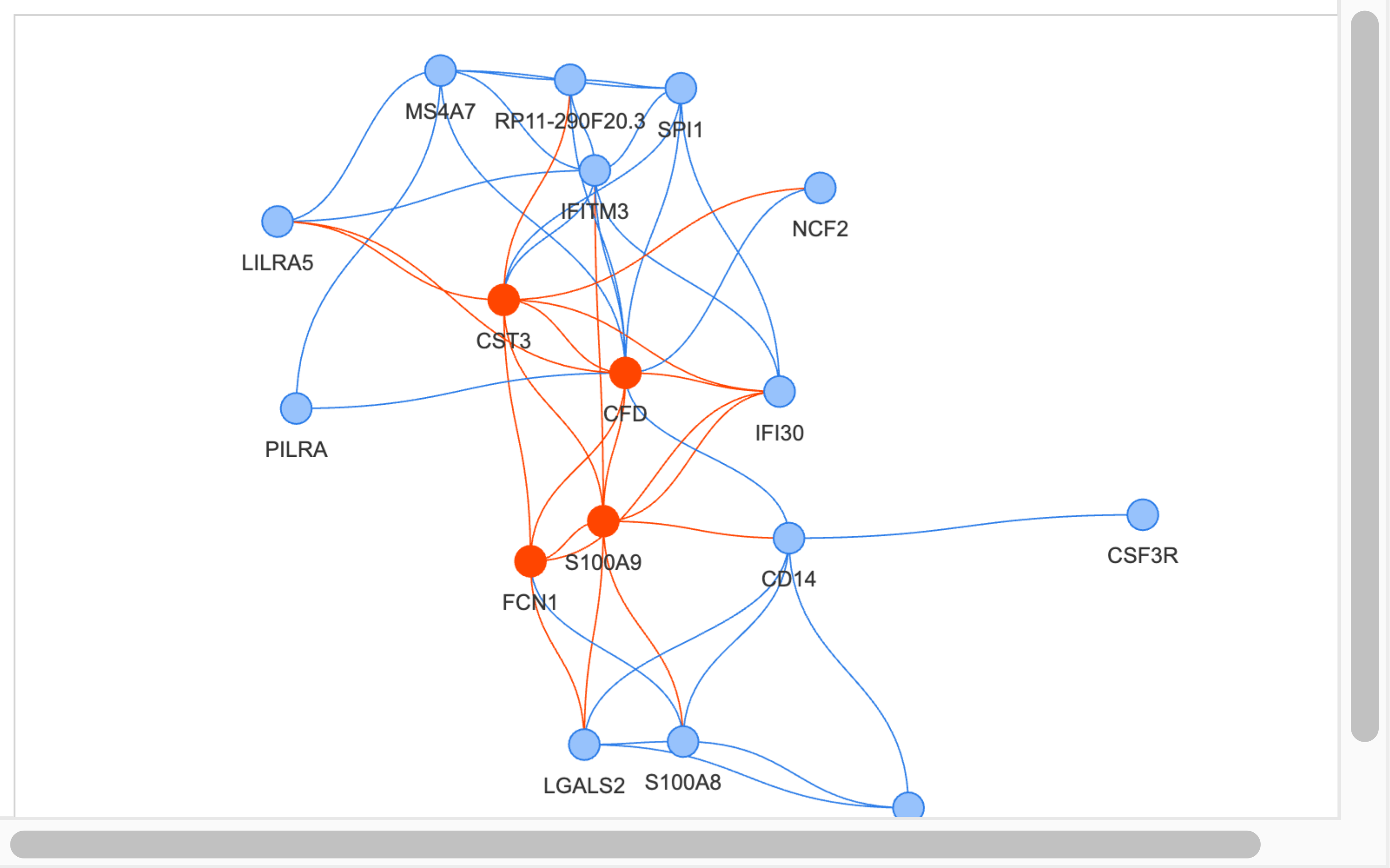

Here we enable the choice to also visualize the drugs targeting the genes within this module.
The drugs have the green stars shape

Options for drug algorithms : "trustrank", "closeness", "degree" ....
The "drug target search" allows to select algorithms that will find genes related to the drugs.
For more information about the algorithms, please refer to [https://drugst.one/doc#implementation_algorithms (https://drugst.one/doc#implementation_algorithms)](https://drugst.one/doc#implementation_algorithms).

```
In [168]: sn.pl.plot_gcn(network_=network_,hub_genes_df=hub_genes_df, name="gcn+drugs", drug_interaction=True, algorit
```

```
Hub genes: ['CFD', 'CST3', 'S100A9', 'FCN1']

INFO:root:trustrank progress is at: 0.0%
INFO:root:trustrank progress is at: 0.0%
INFO:root:trustrank progress is at: 0.0%
INFO:root:trustrank progress is at: 0.0%
INFO:root:trustrank is done.

[['Ribavirin', 'CST3'], ['Methotrexate', 'S100A8'], ['Methyldopa', 'S100A9'], ['Methyldopa', 'S100A8'],
['Dasatinib', 'CSF3R'], ['Lovastatin', 'CD14'], ['Pexidartinib', 'CSF3R'], ['Zinc chloride', 'S100A8'],
['Zinc chloride', 'S100A9'], ['Digoxin', 'CST3'], ['Ruxolitinib', 'CSF3R']]
```

Out[168]:

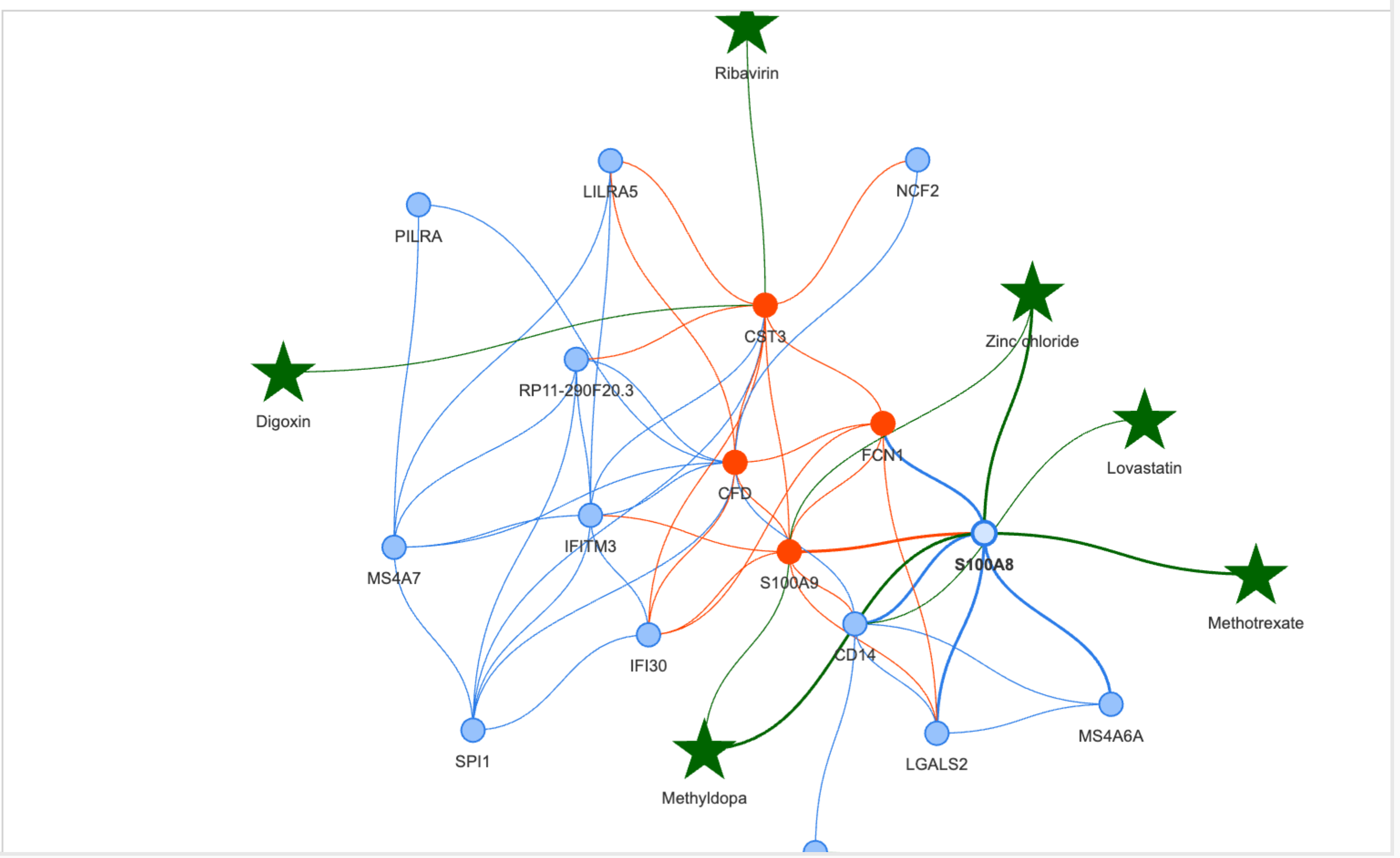

Gene regulatory network inference

```
In [169]: # Load the modules Dataframe
import pandas as pd

modules_df = pd.read_csv("outs-scanet/genes_frequency_df.csv", index_col=0)
modules_df.head()
```

Out[169]:

	Module_r	n_genes	genes	Modules
**0**	yellow	36	["AIM1L", "SSX2IP", "RP11-134G8.7", "SOX13", "...	M1
**1**	tan	59	["STMN1", "RHOC", "CD160", "SH2D2A", "FCRL6", ...	M2
**2**	grey	1528	["TNFRSF4", "ATAD3C", "MMP23B", "CDK11B", "SLC...	M3
**3**	magenta	48	["CA6", "RP3-329E20.2", "PIFO", "AQP10", "SLC1...	M4
**4**	red	38	["SLC2A5", "SLC2A1-AS1", "C1orf177", "RP11-356...	M5

In [170]:
```
# Detect the modules genes that are also transcription factors
# A module with no transcription factors genes cannot be used in the GRN analysis!

specie_ = 'human'
sn.grn.regulators_count(modules_df = modules_df, specie = specie_, figsize=(8,2))
```

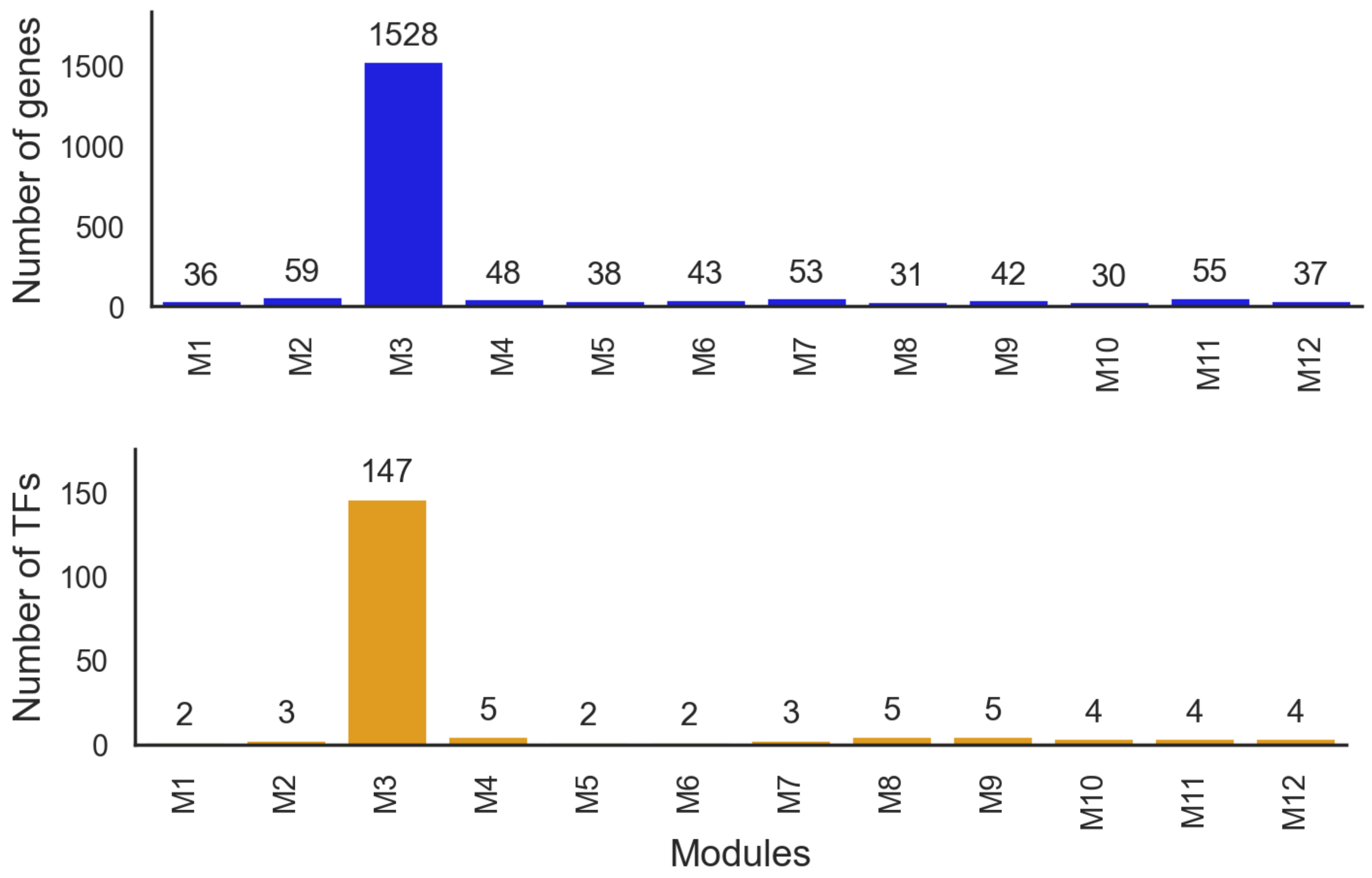

In [171]:
```
#The GRN analysis is done on the full data
# Get all processed data not the reduced one

adata_processed = sn.pp.read_h5ad("data/pbmc3k_raw__processed.h5ad", pr_process="skip")
adata_processed.obs.groupby([cell_annoatation]).size()
```

Out[171]:
```
louvain
CD4 T cells            1110
CD14+ Monocytes         448
B cells                 319
CD8 T cells             307
NK cells                151
FCGR3A+ Monocytes       124
Dendritic cells          18
Megakaryocytes            4
dtype: int64
```

In [172]:
```
# remove all cluster not analyzed !important!

for x in list(set(list(adata_processed.obs[cell_annoatation]))):
    if x not in list(set(list(adata_r.obs["__SCANclusters__"]))):
        adata_processed = adata_processed[adata_processed.obs[cell_annoatation] != x]
adata_processed.obs.groupby([cell_annoatation]).size()
```

Out[172]:
```
louvain
CD4 T cells            1110
CD14+ Monocytes         448
B cells                 319
CD8 T cells             307
NK cells                151
FCGR3A+ Monocytes       124
dtype: int64
```

In [ ]:

GRN analysis parameters
anno_name: cell type or any cell annotation indicating in which cell group where the GRN will be inferences.
groupby_: The key of the observation in the anndata.obs corresponding to the cell clustering to be used.
Mod_: GCN module to be mapped to GRN

In [ ]:

subsampling: The data we are handling is often noisy and sparse despite the preprocessing and filtering. Furthermore, the single-cell data capture a temporal snapshot, where cells are in different states and phases, which leads to cell-to-cell heterogeneity and an abundance of intermediate states. In other words, the gene expression profiles vary even within the same cell type, which is a major obstacle in understanding

the underlying regulatory dynamics within the data. To overcome this, we reasoned that randomly subsampling cells and learning multiple scGRN and combining them into one large scGRN will permit us to eliminate the signals sourced from noise and outlier cells. Through the examination of multiple scGRN, we can distinguish the conserved signals across the sample and the ones from noise. subsampling is an important step to eliminate the noise and therefore accurate scGRN construction, which is crucial for the further steps.

This done using:

n_iteration: Number of time to compute scGRN
subsampling_pct: Percentage of cells to include in every random sub-sample

---

In [ ]:

```

```

A scGRN is computed for each sub-sample resulted from the subsampling step. Next, the sub-sample-based scGRNs are combined into one accurate scGRN. The alignment procedure associates an occurrence rate for every edge, which measures the frequency with which the edge occurs across all scGRNs. Considering that we are interested in capturing the biological insights that are homogeneous within each sample, edges with low occurrence rates mostly emerge from outlier or Inferior sets of cells, and those are to be filtered out. The remaining edges compose the final sample-based scGRN.

one can set an occurrence threshold based on this rate to filter the low confidence edges. We recommend 80%.

In [ ]:

```

```

In [173]:

```
print(anno_name_)
```

```
['CD14..Monocytes']
```

In [174]:

```
# GRN analysis parameters

anno_name = "CD14+ Monocytes"
groupby_ = cell_annoatation
Mod_ = M
num_workers = 60
n_iteration = 10
```

Get the ranking databases
See for more details : [https://pyscenic.readthedocs.io/en/latest/tutorial.html (https://pyscenic.readthedocs.io/en/latest/tutorial.html)](https://pyscenic.readthedocs.io/en/latest/tutorial.html)

In [176]:

```
sn.download_db()
```

In [177]:

```
# GRN inference

grn_df = sn.grn.grn_inference(adata_processed=adata_processed,modules_df=modules_df, module=Mod_, groupby_=g
```

```
This Module has 55 genes.
4 Genes in this modules are regulatores (TFs).
TFs are: ['KLF4', 'HHEX', 'SPI1', 'POU2F2']
The anndata size after gene filtring (2459, 55) ...
Using anootation : CD14+ Monocytes found 448 cells.
runing : 10 iteration ...
/tmp/tmpkbw1my82/giadmgjacn_adata.loom
Subsampling using 80% ...
Using 358 cells after sub-sampling ...
/tmp/tmpkbw1my82/szyiyuthrx_adata.loom
Subsampling using 80% ...
Using 358 cells after sub-sampling ...
/tmp/tmpkbw1my82/xzmuhyjapb_adata.loom
Subsampling using 80% ...
Using 358 cells after sub-sampling ...
/tmp/tmpkbw1my82/bxpuzvfwrb_adata.loom
Subsampling using 80% ...
Using 358 cells after sub-sampling ...
/tmp/tmpkbw1my82/eyshxibair_adata.loom
```

In [178]:

```python
# GRN
#one can set an occurrence threshold based on this rate to filter the low confidence edges.
#We recommend 80%.

print(grn_df.shape)
grn_df = grn_df.sort_values(by=['occurrence(pct)'], ascending=False)
grn_df.head(6)
# Save for later use
# grn_df.to_csv('outs/grn_df.csv')
```

```
(67, 3)
```

Out[178]:

	TF	TG	occurrence(pct)
**33**	SPI1	APOBEC3A	100.0
**34**	SPI1	APOBEC3B	100.0
**64**	SPI1	TMEM176B	100.0
**63**	SPI1	SPI1	100.0
**61**	SPI1	PLBD1	100.0
**60**	SPI1	PILRA	100.0

Network visualization

GRN visualization
The TF (orange triangles) and their target genes one can visualize the whole network or just select regulon(s) (a TF and its target genes) of interest in case of huge networks using [regulon] parameter.

In [179]:

```python
sn.pl.plot_grn(df=grn_df, occurrence_pct=40, name="GRN_net", regulon="all", layout="None")
```

```
 Out of 67 edges, 37 edges satisfying occurrence threshold 40% where kept

['KLF4', 'SPI1']
(37, 3)
None
```

Out[179]:

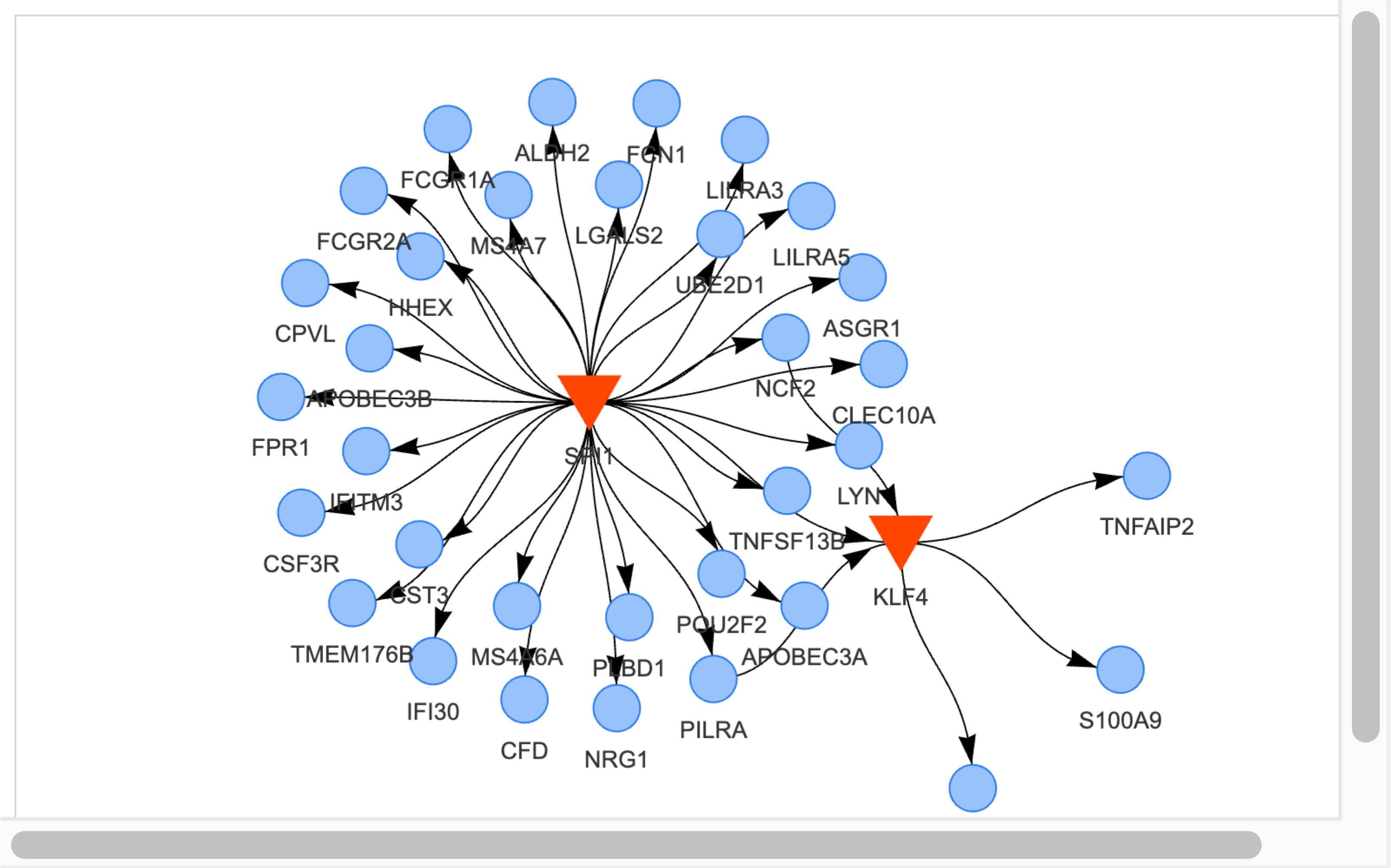

Here we enable the choice to also visualize the drugs targeting the genes within this module.
The drugs have the green stars shape

In [180]:
```python
sn.pl.plot_grn(df=grn_df, occurrence_pct=80, regulon="all", layout="None", drug_interaction="direct")
```

```
 Out of 67 edges, 24 edges satisfying occurrence threshold 80% where kept

['SPI1']
(24, 3)
None

INFO:root:degree progress is at: 0.0%
INFO:root:degree progress is at: 0.0%
INFO:root:degree progress is at: 0.0%
INFO:root:degree progress is at: 0.0%
INFO:root:degree progress is at: 66.66666666666666%
INFO:root:degree is done.
```

Out[180]:

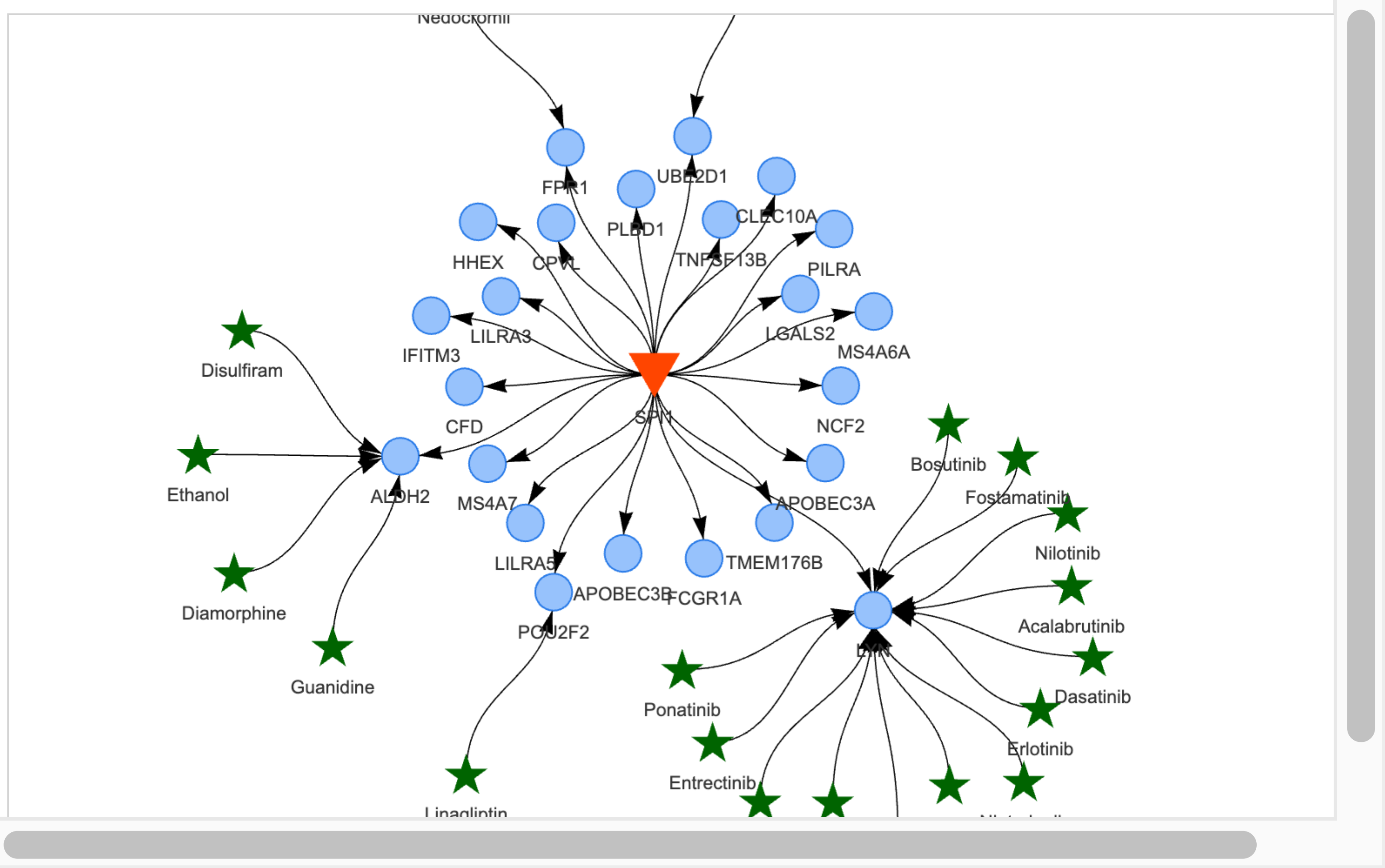

In [ ]:
